# Genomic Alteration Characterization in Colorectal Cancer Identifies a Prognostic and Metastasis Biomarker: FAM83A|IDO1

**DOI:** 10.3389/fonc.2021.632430

**Published:** 2021-04-20

**Authors:** Zaoqu Liu, Yuyuan Zhang, Qin Dang, Kunpeng Wu, Dechao Jiao, Zhen Li, Zhenqiang Sun, Xinwei Han

**Affiliations:** ^1^Department of Interventional Radiology, The First Affiliated Hospital of Zhengzhou University, Zhengzhou, China; ^2^Interventional Institute of Zhengzhou University, Zhengzhou, China; ^3^Interventional Treatment and Clinical Research Center of Henan Province, Zhengzhou, China; ^4^Department of Colorectal Surgery, The First Affiliated Hospital of Zhengzhou University, Zhengzhou, China

**Keywords:** genomic alteration, mutational signature, molecular subtype, colorectal cancer, prognosis, metastasis

## Abstract

Genomic alterations constitute crucial elements of colorectal cancer (CRC). However, a comprehensive understanding of CRC genomic alterations from a global perspective is lacking. In this study, a total of 2,778 patients in 15 public datasets were enrolled. Tissues and clinical information of 30 patients were also collected. We successfully identified two distinct mutation signature clusters (MSC) featured by massive mutations and dominant somatic copy number alterations (SCNA), respectively. MSC-1 was associated with defective DNA mismatch repair, exhibiting more frequent mutations such as *ATM, BRAF*, and *SMAD4*. The mutational co-occurrences of *BRAF*-*HMCN* and *DNAH17*-*MDN1* as well as the methylation silence event of MLH-1 were only found in MSC-1. MSC-2 was linked to the carcinogenic process of age and tobacco chewing habit, exhibiting dominant SCNA such as *MYC* (8q24.21) and *PTEN* (10q23.31) deletion as well as *CCND3* (6p21.1) and *ERBB2* (17q12) amplification. MSC-1 displayed higher immunogenicity and immune infiltration. MSC-2 had better prognosis and significant stromal activation. Based on the two subtypes, we identified and validated the expression relationship of *FAM83A* and *IDO1* as a robust biomarker for prognosis and distant metastasis of CRC in 15 independent cohorts and qRT-PCR data from 30 samples. These results advance precise treatment and clinical management in CRC.

## Introduction

Colorectal cancer (CRC) is the fourth most prevalent cancer and the second most lethal cancer globally (1). Although considerable improvements in surgical techniques and chemoradiotherapy have extended overall survival (OS) for many patients, the mortality of CRC remains high (2). Recently, immunotherapy has made tremendous progress and achieved clinical success in CRC, but only a small proportion of patients benefit (3). Hence, it is imperative to improve individualized treatment and clinical management in CRC.

For decades, the TNM and Dukes classification schemes have been valuable for assessing the prognosis of, and selecting treatment for, CRC patients (4). However, accumulating evidence indicates that CRC patients with the same stage present diverse biological behaviors and clinical outcomes because of high heterogeneity (5). Thus, these conventional criteria fail to meet the needs of precision treatment in CRC. With advances in the molecular biological understanding of CRC, the CRC Subtyping Consortium proposed four consensus molecular subtypes (CMSs) in 2015 (6). The CMS classification can help guide clinical treatment and evaluate prognosis. For example, CMS4, characterized by epithelial–mesenchymal transition (EMT) and primary resistance to anti-EGFR therapy, has a poor prognosis relative to other subtypes, whereas CMS3, linked to metabolic reprogramming and altered cellular metabolism, displays favorable survival (6, 7). Of note, the CMS classification only considers a fraction of genomic variations, such as *BRAF, TP53*, and *KRAS* mutations, *HNF4A* amplification, and homozygous deletion of *PTEN*, but there is a wide range of genomic variations in CRC (6–13); thus, the genomic variations considered in the CMS classification system cannot fully explain the molecular heterogeneity of CRC, and the CMS classification system might ignore a large number of potential therapeutic targets and genomic drivers. Thus, it is necessary to systematically explore the heterogeneity of CRC based on global genomic variations and further provide references for optimizing targeted treatment of CRC patients.

Currently, 30 mutational signatures, which can be attributed to specific causes of DNA lesions, such as defective DNA repair and exogenous or endogenous mutagen exposure, have been summarized by previous research (14). Regrettably, the mutational signatures of CRC have not been dissected in detail until now. In addition, CRC possesses an extremely high number of genomic variations (7), some of which play a vital role in predicting prognosis and guiding treatment. A previous study demonstrated that colon cancer patients harboring the same *BRAF*^*V*600*E*^ oncogenic lesion generally displayed a low median survival (15). A randomized, phase III trial indicated that patients with wild-type *RAS* were sensitive to anti-EGFR therapy; conversely, patients with *KRAS* mutations displayed resistance to anti-EGFR therapy (16). In addition, CRC patients with the *LMNA–NTRK1* gene fusion were sensitive to the *TRKA* kinase inhibitor entrectinib (17). In addition to these genomic variations that have been proven to be predominantly associated with prognosis and targeted therapies in CRC, there are still many genomic variations that might have clinical significance waiting to be discovered.

In this research, we systematically extracted eight mutational signatures of CRC. Based on these mutational signatures, we performed consensus clustering to recognize heterogeneous molecular subtypes and better understand the genomic characteristics of CRC. Ultimately, we identified two distinct mutational signature clusters (MSCs). The two subtypes displayed significant difference in the genomic variation, methylation profile, prognosis, immune landscape, and microsatellite instability (MSI) status. In addition, based on the two subtypes, we identified and validated the relationship between *FAM83A* and *IDO1* expression as a promising biomarker for predicting prognosis and distant metastasis in CRC patients in 15 independent public datasets and qRT-PCR data from 30 samples. These results deepen the understanding of the heterogeneity of CRC and will facilitate the individualized treatment and clinical management of patients with CRC.

## Materials and Methods

### Data Sources and Processing

Mutation (derived from VarScan 2), somatic copy number alteration (SCNA), HumanMethylation450 array, and RNA-seq count data of CRC were obtained from The Cancer Genome Atlas (TCGA) portal. We also retrieved 14 expression microarrays datasets (GSE17536, GSE17537, GSE103479, GSE29621, GSE38832, GSE39084, GSE39852, GSE71187, GSE72970, GSE87211, GSE27854, GSE21510, GSE18105, and GSE71222) from the Gene Expression Omnibus (GEO) database. For 11 microarray datasets derived from the Affymetrix Human Genome U133 Plus 2.0 Array, the “CEL” raw data were obtained and further processed via a robust multiarray averaging algorithm (RMA) implemented in the affy R package. RMA was used to perform background adjustment, quantile normalization, and final summarization of oligonucleotides per transcript using the median polish algorithm. For the three other microarray datasets, we directly retrieved the normalized matrix files. The corresponding clinical information was also downloaded, and the details are listed in Supplementary Table 1. Ultimately, a total of 2,778 patients' data were collected, of whom 2,294 patients had survival information and 1,144 patients had metastasis information.

### Deciphering Mutational Signatures in Colorectal Cancer

The masked somatic mutational profiles of 535 CRC patients were obtained from TCGA datasets. The trinucleotideMatrix function of the maftools package (18) was employed to extract the 5′ and 3′ bases immediately flanking the mutated site and to then generate a 96 × 535 mutation subtype frequency matrix. Subsequently, we applied the NMF package to extract the mutational signature, and the optimal rank was determined by the cophenetic coefficient and the residual sum of squares (RSS). *De novo* mutational signatures were then compared with curated signatures in COSMIC (19) using cosine similarity analysis (20) (https://cancer.sanger.ac.uk/cosmic/signatures_v2). The APOBEC enrichment analysis described by Roberts et al. (21) was further performed with the Maftools package.

### Consensus Clustering

Based on the extracted mutational signatures, consensus clustering was utilized to determine the membership of CRC patients within possible clusters using the ConsensusClusterPlus R package (22). The subsample is 80% of the samples at each iteration, and each subsample was partitioned up to k (max K = 9) groups by k-means algorithm upon the Euclidean distance. This process was repeated 1,000. The optimal number of clusters was determined by cumulative distribution function (CDF) and proportion of ambiguous clustering (PAC) analyses (23). In addition, the Nbclust (24) package, which provides 26 indices in determining the number of clusters, was also used to assess the best clustering scheme.

### Mutation and Somatic Copy Number Alteration Analysis

The MutSigCV 1.4 software (25) was employed to identify the significantly mutated genes (SMGs) for the two MSC subtypes of CRC. Genes with q < 0.05 and mutation frequencies > 10% were defined as mutation drivers. The GISTIC2.0 software was employed to identify significantly altered segments. Fragments with q < 0.05 and alteration frequency > 25% were considered SCNA drivers. The load of loss or gain was quantified as the total number of all genes with SCNA at the focal and arm levels. Mutations and SCNAs in MMR genes, including *MLH1, MLH3, MSH2, MSH3, MSH4, MSH5, MSH6, PMS1*, and *PMS2*, were also explored in the two MSC subtypes.

### Identification of Methylation-Driven Genes

We downloaded the HumanMethylation450 array data from the TCGA-CRC cohort and employed the IlluminaHumanMethylation450kanno.ilmn12.hg19 package for annotation. To identify the methylation-driven genes (MDGs) in CRC, we employed two methods to dissect the methylation profiling data. One method was MethylMix, which is based on the beta distribution and was designed to recognize gene expression that is significantly related to methylation events (26). The other method was derived from the study of Charoentong et al. (27) and can be used to identify epigenetically silenced genes according to the absolute expression difference between the methylation and unmethylation groups. The MDGs were ultimately determined by the intersection of the two methods. In addition, if one MDG had a dominant difference in both the mRNA expression and DNA methylation profile between the two MSC subtypes (*p* < 0.05), we then labeled it a subtype-specific MDG (ssMDG).

### Functional Annotation and Immune-Related Indicator Assessment

We performed gene set enrichment analysis (GSEA) between the two MSC subtypes, and the biological function terms with FDR < 0.05 were considered significant. Fifty hallmark pathways were also retrieved from the Molecular Signature Database (MSigDB v7.1). Based on the hallmark gene sets, we utilized the gene set variation analysis (GSVA) algorithm to transform the gene expression matrix into a pathway enrichment score matrix. The limma R package was applied to further reveal the discrepant pathways between the two MSC subtypes, with thresholds of FDR < 0.05 and |log2FC| > 0.2. The abundances of eight immune cell and two non-immune cell populations were assessed via the MCP-counter R package. In addition, we also calculated or retrieved data for 17 immunogenicity indicators from previous research: non-silent mutation rate, MSI score, single nucleotide variant (SNV) neoantigens, insertion and deletion (indel) neoantigens, cancer testis antigen (CTA) score, aneuploidy score (AS), intratumor heterogeneity (ITH) score, number or fraction of segments altered, homologous repair deficiency (HRD) score, loss of heterozygosity (LOH) score, B-cell receptor (BCR) or T-cell receptor (TCR) diversity score, and cytolytic activity (CYT) (28–30). The antigen processing and presenting machinery score (APS), used to measure antigen presentation capacity, was further calculated according to a previous report (31). For details on the immune-related indicators, please refer to Supplementary Table 2. Moreover, multiomics events of 75 immunomodulator molecules were further analyzed (Supplementary Table 3), including somatic mutations, SCNAs, and DNA methylation (28). The FDR was calculated with the Benjamini–Hochberg multiple correction method.

### Identification of Reliable Gene Pair Markers for Prognosis and Distant Metastasis

We aimed to identify the relationship between the mRNA expression of two genes with prognostic significance and utility for predicting distant metastasis to facilitate clinical management. To ensure the robustness and stability of our results, 11 independent CRC cohorts with complete prognostic information encompassing (TCGA-CRC, GSE17536, GSE17537, GSE103479, GSE29621, GSE38832, GSE39084, GSE39852, GSE71187, GSE72970, and GSE87211) were employed to identify promising prognostic markers, and seven independent CRC cohorts with distant metastasis information (TCGA-CRC, GSE39084, GSE29621, GSE27854, GSE21510, GSE18105, and GSE71222) were utilized to further explore the ability of the identified prognostic markers to predict metastasis (Supplementary Table 1). The pipeline was as follows: (1) The edgeR package, with criteria |log2FC| > 1 and FDR < 0.05, was applied to identify differentially expressed genes (DEGs) between the MSC subtypes in the TCGA-CRC cohort. (2) Based on the identified subtype-specific DEGs, we converted the mRNA expression matrix into a two-gene expression relationship matrix. For one gene pair A|B, if the expression of A was greater than that of B, the pair was labeled “A>B”; conversely, if the expression of B was greater than that of A, the pair was labeled “B>A.” If the expression of A was equal to B, the sample was discarded. If the proportion of samples with “A>B” or “B>A” was > 90% in the corresponding cohort, the gene pair was discarded. (3) Univariate Cox regression analysis was implemented to screen the gene pairs with significant prognostic value (FDR < 0.05) in each cohort. If one gene pair had an FDR < 0.05 in more than five cohorts, then it was defined as a consensus prognosis gene pair signature (CPGPS). (4) We further explored the ability of each CPGPS to predict metastasis in seven independent cohorts with metastasis information.

### Human Colorectal Cancer Specimens

The use of the human cancer tissues in this study was approved by the Ethics Committee of The First Affiliated Hospital of Zhengzhou University on December 19, 2019, and the TRN is 2019-KW-423. A total of 30 paired CRC tissues and matched adjacent non-tumor tissues were obtained from patients after receipt of surgical resection at The First Affiliated Hospital of Zhengzhou University. None of the patients received any preoperative chemotherapy or radiotherapy. Written informed consent was obtained from all patients. The inclusion criteria were as follows: no preoperative chemotherapy, radiotherapy, or targeted therapy; no other types of tumors; and no autoimmune diseases. The specimens obtained during surgery were immediately snap frozen in liquid nitrogen and stored at −80°C until RNA extraction. Clinical staging of the specimens was based on the NCCN (2019) guidelines. For details on the patients, please refer to Supplementary Table 4.

### RNA Preparation and Quantitative Real-Time PCR

Total RNA was isolated from CRC tissues and paired adjacent non-tumor tissues with RNAiso Plus reagent (Takara, Dalian, China) according to the manufacturer's instructions. RNA quality was evaluated using a NanoDrop One C (Waltham, MA, USA), and RNA integrity was assessed using agarose gel electrophoresis. An aliquot of 1 μg of total RNA was reverse transcribed into complementary DNA (cDNA) according to the manufacturer's protocol using a High-Capacity cDNA Reverse Transcription kit (TaKaRa BIO, Japan). Quantitative real-time PCR (qRT-PCR) was performed using SYBR Assay I Low ROX (Eurogentec, USA) and SYBR® Green PCR Master Mix (Yeason, Shanghai, China) to detect expression. The data were normalized to the expression of *GAPDH*. The sequences of the primers were as follows:

*GAPDH* forward (5′- to 3′-): GGAGCGAGATCCCTCCAAAAT
*GAPDH* reverse (5′- to 3′-): GGCTGTTGTCATACTTCTCATGG
*FAM83A* forward (5′- to 3′-): CAGATCTCTGACAGTCACCTCAAG
*FAM83A* reverse (5′- to 3′-): CTGCCTGACTTGGCACAGTA
*IDO1* forward (5′- to 3′-): ATATGCCACCAGCTCACAGG
*IDO1* reverse (5′- to 3′-): AGCTTTCACACAGGCGTCAT.

### Statistical Analysis

Correlations between two continuous variables were assessed via Spearman's correlation coefficients. Fisher's exact test or Pearson's chi-squared test was applied to compare categorical variables. Continuous variables were compared between two groups through the Wilcoxon rank-sum test or *T-*test. The Wilcoxon signed rank test was utilized to compare the gene expression differences between the paired CRC tissues and matched adjacent non-tumor tissues in the qRT-PCR assay. Kaplan–Meier and Cox regression analyses were performed with the survival R package. All *P-*values were two-sided, with *p* < 0.05 considered to indicate statistical significance. All data processing, statistical analysis, and plotting were conducted in R 3.6.4 software.

## Results

### Extraction of Mutational Signatures in Colorectal Cancer

**Figure 1:**
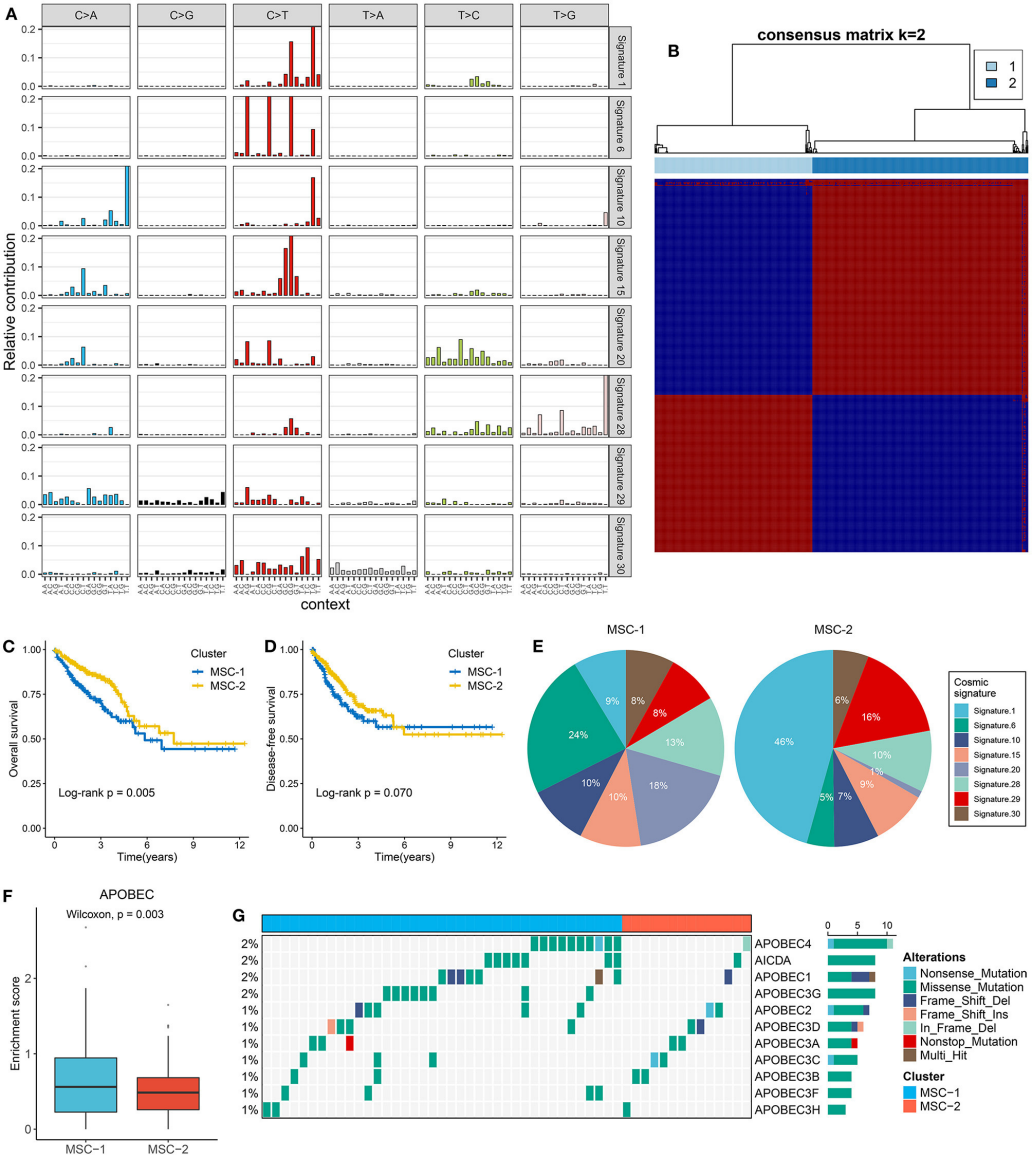
The extraction of mutation signatures and generation of the mutation signature relevant subtypes in CRC. **(A)** Eight mutation signatures were deciphered (mutation signature 1, 6, 10, 15, 20, 28, 29, and 30) based on NMF algorithm and COSMIC signatures. **(B)** The consensus score matrix of all samples when k = 2. A higher consensus score between two samples indicates they are more likely to be grouped into the same cluster in different iterations. The rows and columns of heatmap are both samples, and the cell value of the heatmap is the consensus score between two samples. **(C,D)** Kaplan–Meier analysis for OS **(C)** and DFS **(D)** between MSC-1 and MSC-2. **(E)** Pie charts show the relative proportion of eight categories of mutation patterns in MSC-1 and MSC-2, respectively. **(F)** The difference of *APOBEC* enrichment score between MSC-1 and MSC-2. **(G)** Mutational oncoplot of 11 *APOBEC* associated genes in two subtypes.

A total of 192,905 mutations were detected in 535 samples, with a median of 91 mutations per sample, including SNVs and small indels. SNVs were the main mutation type, and C>T mutations displayed the highest frequency, followed by C>A and T>C mutations. Among the top 10 most frequently mutated genes, *APC* had the highest number of deletion mutations (236), and *TTN* had the highest number of missense mutations (646) (Supplementary Figure 1). To gain insights into the potential mutation generation processes occurring in patients with CRC, we deconvoluted the mutational signatures via the NMF algorithm (Supplementary Figure 2A). Subsequently, eight mutational signatures were extracted from the CRC genomic data and annotated with the COSMIC signature nomenclature based on cosine similarity (Supplementary Figure 2B). Therefore, the extracted mutational signatures were ultimately called cosmic signatures 1, 6, 10, 15, 20, 28, 29, and 30 (Figure 1A). Clocklike signature 1 is thought to be connected with the age-related accumulation of spontaneous deamination of 5-methylcytosine. Signatures 6, 15, and 20 are all associated with defective DNA MMR. Signature 10 features altered the activity of the error-prone polymerase *POLE* and is often found in six cancer types, including CRC. Signature 29 exhibits transcriptional strand bias for C>A mutations due to tobacco chewing habits. Signatures 28 and 30 have been observed in a subset of stomach and breast cancers with unknown etiology (19).

### Generation of the Mutational Signature-Related Subtypes

Mutational signatures were deciphered from CRC genome data, and consensus clustering analysis revealed that two was the optimal number of subtypes (Figure 1B). The CDF curve, PAC value, and Nbclust results further confirmed the stability and reliability of the cluster results (Supplementary Figures 2C–E). We annotated the two subtypes as mutational signature clusters (MSCs) 1 and 2. The Kaplan–Meier survival analysis suggested that MSC-2 was significantly associated with favorable OS (log-rank *p* = 0.005) (Figure 1C). There was no significant difference in disease-free survival (DFS) between the two subtypes, possibly due to the large amount of follow-up data censored after 5 years (log-rank *p* = 0.070) (Figure 1D). We further compared the differences in DFS between the two subtypes within 5 years and found that MSC-2 was significantly associated with better DFS (log-rank *p* = 0.028) (Supplementary Figure 2F). Of note, MSC-1 (*n* = 226) predominantly featured signatures 6, 15, 20, and 28. Signatures 6, 15, and 20 are linked to defective DNA MMR. MSC-2 (*n* = 309) was characterized by signatures 1 and 29, which are associated with age and tobacco chewing habits (Figure 1E, Supplementary Figure 2G). We also observed that the *APOBEC* signature enrichment score was significantly higher (*p* = 0.003) in MSC-1, which indicated that MSC-1 had a higher occurrence of the C>T transition in TpCpW motifs than did MSC-2 (Figure 1F). A previous study demonstrated that mutations in the *APOBEC* family might contribute to a high tumor mutation burden (TMB) (32). Therefore, we further explored mutations in the *APOBEC* family and found rare mutations in CRC patients. Of note, MSC-1 had a higher frequency of mutations than MSC-2, in line with the enrichment score of *APOBEC* mutations found in MSC-1 compared with MSC-2 (*p* = 0.003) (Figure 1G); this finding might explain the high mutation rate in MSC-1.

### Somatic Mutation Landscape of the Two Subtypes

**Figure 2:**
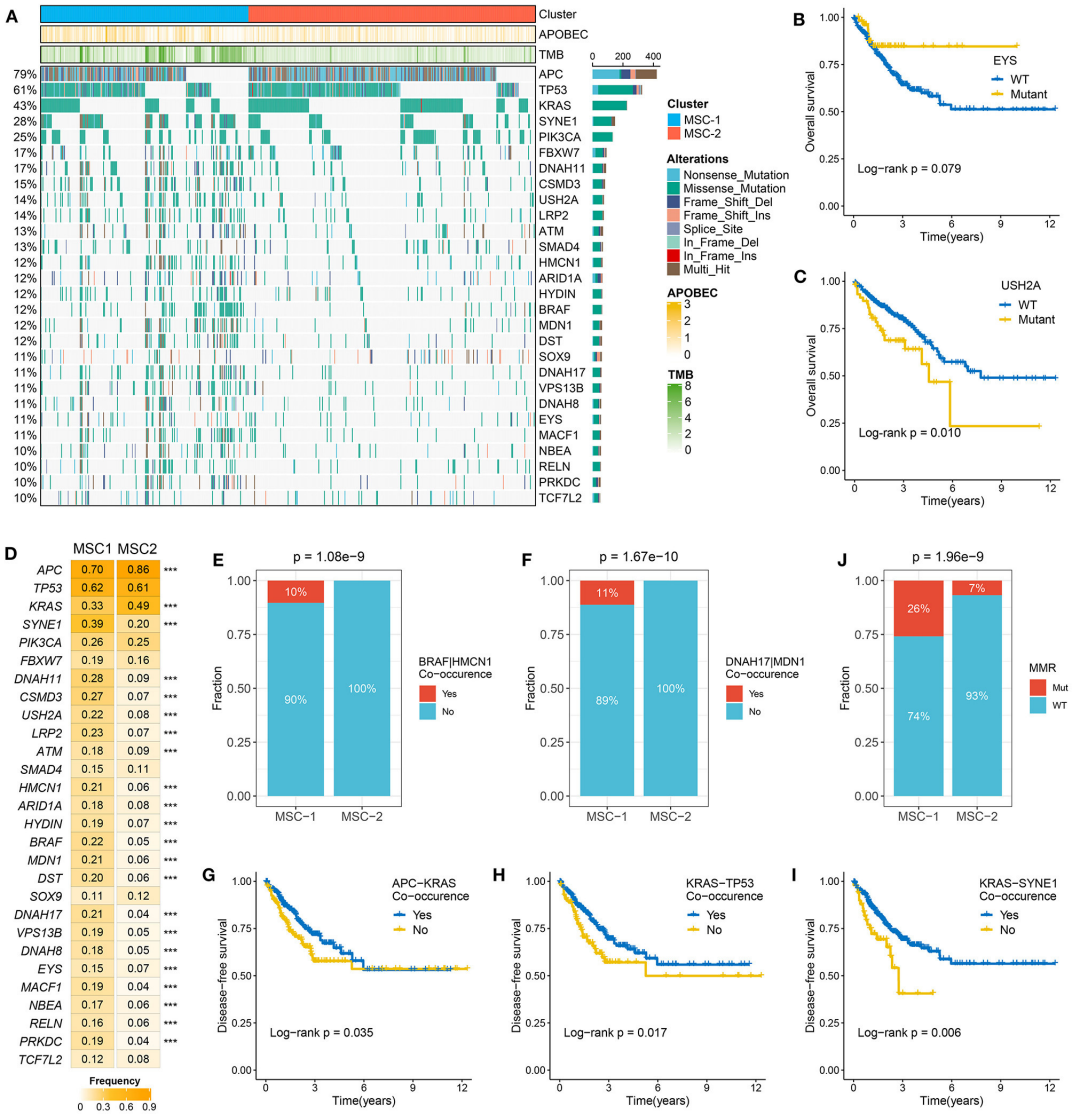
The mutation driven genes in colorectal cancer (CRC). **(A)** Mutational oncoplot of mutation driven genes in MSC-1 and MSC-2. Rows are genes and columns are tumor samples. **(B,C)** Kaplan–Meier survival analysis of *EYS* **(B)** and *USH2A* mutations **(C)**. **(D)** The mutation frequency of mutational drivers in two subtypes, ***P < 0.001. **(E,F)** The relative proportion of *BRAF-HMCN* **(E)** and *DNAH17-MDN1* **(F)** co-occurrences in two subtypes. **(J)** The relative proportion of patients with the MMR genes mutations in two subtypes. **(G–I)** Kaplan–Meier survival analysis of *APC–KRAS* **(G)**, *KRAS–TP53* **(H)**, and *KRAS–SYNE1* **(I)** co-occurrence.

The TMB in MSC-1 cells was significantly higher than that in MSC-2 cells (*p* < 0.001) (Supplementary Figure 3A). A higher TMB may tend to occur in patients with defective DNA MMR-related mutational signatures (7). We further determined 28 mutation-driven genes with MutSigCV q < 0.05 and mutation frequencies > 10% in CRC (Supplementary Table 5, Figure 2A). Out of these 28 genes, 18 genes have been reported in at least one CRC-associated study, such as *APC, TP53, KRAS, SYNE1, PIKSCA, FBXW7*, etc. (33–39). In addition, 10 novel drivers were identified: *DNAH11, USHA2, HMCN1, HYDIN, MDN1, DST, VPS13B, DNAH8, EYS*, and *NBEA*. We also determined the prognostic role of these genes. Mutation of *EYS* prolonged DFS, and mutation of *USH2A* suggested unfavorable OS (Figures 2B,C). In the two MSC subtypes, 22 out of the 28 drivers exhibited significant mutation differences (Figure 2D). Consistent with the high TMB in MSC-1, the mutation frequency of most drivers, such as *ATM, SOX-9*, and *PRKDC*, was also higher in MSC-1 than in MSC-2. Of note, *APC* and *KRAS*, genes that are mutated early in colon adenoma–carcinoma progression (40), were dominantly mutated in MSC-2, which implies that familial adenomatous polyposis (FAP) may be one of the main causes of MSC-2. Further analyses revealed predominant commutations of *KRAS* and *SYNE1, TP53* and *SYNE1*, and *APC* and *USH2A* (Supplementary Figure 3B). Interestingly, we found some specific commutations, such as BRAF-*HMCN* and *DNAH17-MDN1*, that appeared only in MSC-1, which suggested that these commutations could be employed to distinguish different subtypes (*BRAF-HMCN*: *p* < 0.001; *DNAH17-MDN1*: *p* < 0.001) (Figures 2E,F). In addition, for the first time, we determined the prognostic value of some commutations: commutation of *APC-TP53* demonstrated favorable DFS (Supplementary Figure 3C) and commutations of *APC-KRAS, KRAS-TP53*, and *KRAS-SYNE1* were significantly associated with poor DFS (Figures 2G–I). Furthermore, the literature has confirmed that CRC patients with defective mismatch repair (MMR) can develop hypermutation and MSI (41). Hence, we investigated the mutation status of nine known MMR genes, and the results showed that MSC-1 had the most mutations in MRR genes (Supplementary Figure 3D), and the proportion of cases with MMR gene mutations was relatively high in MSC-1 compared with MSC-2 (26 vs. 7%; *p* < 0.001) (Figure 2J); these results were in line with the specific mutational signatures in MSC-1, such as signatures 6, 15, and 20.

### Somatic Copy Number Alterations Investigation of the Two Subtypes

**Figure 3:**
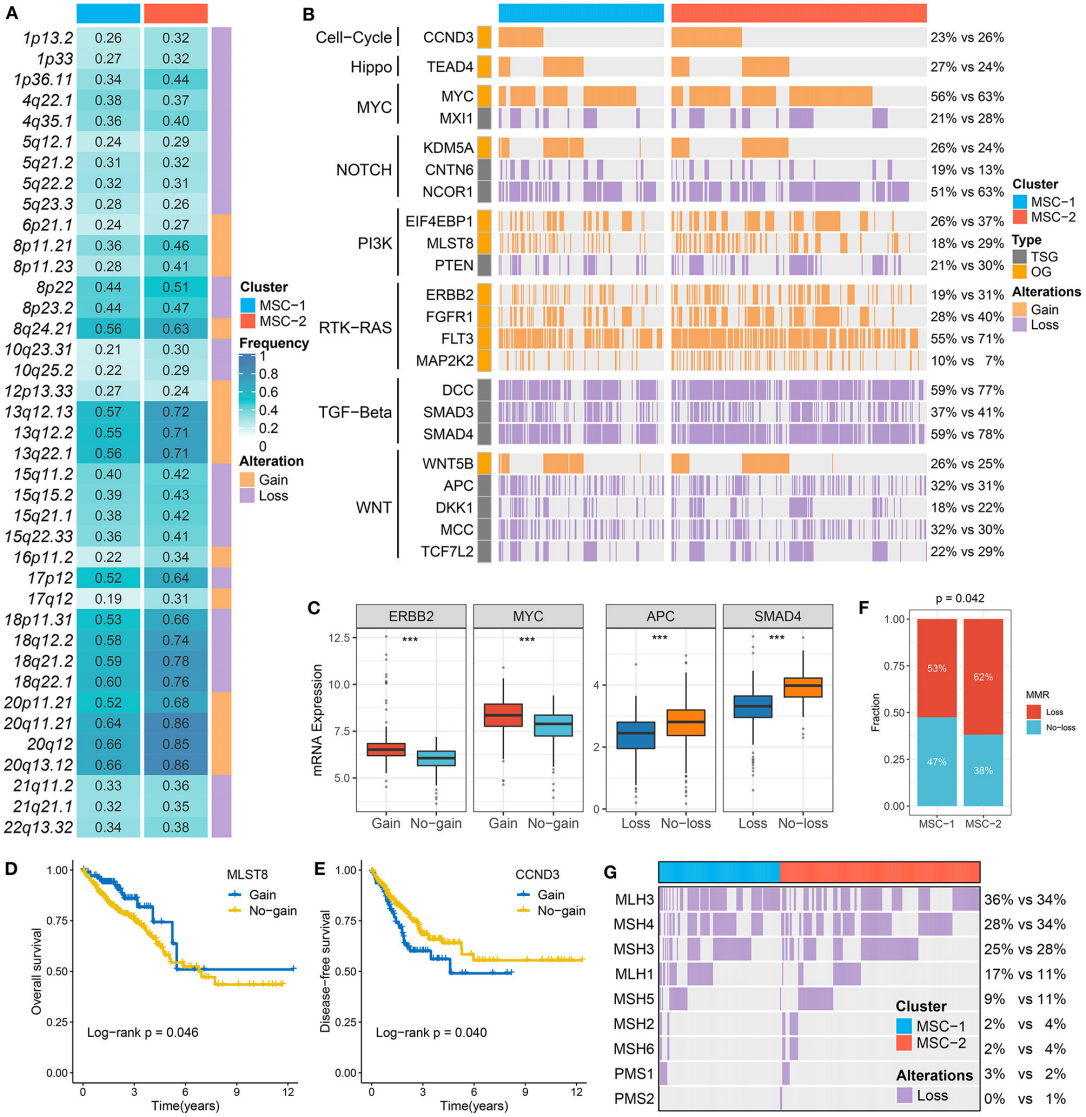
The driven segments identified from GISTIC algorithm in CRC. **(A)** The amplification (orange) and deletion (purple) frequency of 39 driven segments in two subtypes. **(B)** The distribution of CNA relevant oncogenes and tumor suppressive genes in two subtypes. **(C)** The expression difference of *ERRB2* and *MYC* between the gain and no-gain groups, as well as *APC* and *SMAD4* between the loss and no-loss groups. ***P < 0.001. **(D,E)** Kaplan–Meier survival analysis of *MLST* **(D)** and *CCND3* **(E)** gain. **(F)** The relative proportion of patients with the MMR genes deletions in two subtypes. **(G)** Oncoplot for the deletion of nine MMR-related genes in two subtypes.

At the arm level, both the gain and loss loads were significantly higher in MSC-2 than in MSC-1 (*p* < 0.05). Although there was no statistical significance in the focal level load between the two subtypes, slight trends for higher loads were shown in MSC-2 than in MSC-1 (Supplementary Figure 4A). In contrast to MSC-1, which was characterized by a high mutation load, MSC-2 might predominantly contain alterations in copy number. By employing the GISTIC algorithm, we ultimately identified 39 driver segments encompassing 14 amplification segments and 25 deletion segments (Supplementary Tables 6, 7, Supplementary Figure 4B). We further compared the alteration frequencies of the 39 segments between the two subtypes and found that MSC-1 had a generally low frequency compared with MSC-2, in accordance with the CNA load (Figure 3A). We also found a multitude of oncogenes and tumor suppressor genes in these driver segments that might play an essential role in the tumorigenesis and progression of CRC, such as *MYC* (8q24.21), *CCND3* (6p21.1), *ERBB2* (17q12), *PTEN* (10q23.31), *SMAD4* (18q21.2), and *APC* (5q22.2) (Figure 3B). Although MSC-2 generally had frequent SCNA events involving these genes, high proportions of amplifications or deletions still occurred in MSC-1, involving genes such as *MYC, FTK3*, and *MCC* as well as *NOTCH* and *TGF-beta* pathway-associated genes. Interestingly, we found oncogenes with only amplification and tumor suppressor genes with only deletion. Thus, the gene expression differences between gain and no-gain mutations, and loss and no-loss mutations were further explored, and we found that oncogenes with gain, such as *ERBB2, MYC*, and *MLST8*, were more prone to overexpression, and the expression of tumor suppressor genes with loss was predominantly lower than that of tumor suppressor genes with no-loss, such as *APC, SMAD4*, and *PTEN* (*p* < 0.001) (Figure 3C, Supplementary Figure 4C). These results suggest that CNA status plays a master regulatory role in the aberrant expression of oncogenes and tumor suppressor genes in CRC. Further survival analysis demonstrated the prognostic significance of these genes (Supplementary Figures 4D,E). We report for the first time that gain of *MLST8* and *MAP2K2* prolonged OS (Figure 3D, Supplementary Figure 4F), gain of *CCND3* indicated worse DFS (Figure 3E), and loss of *CTNN6, DKK1, APC, MCC*, or *SMAD4* was associated with unfavorable DFS (Supplementary Figure 4F). Moreover, we also investigated the CNA of MMR genes and found that the fraction of patients with MMR gene deletions was higher in MSC-2 than in MSC-1 (62 vs. 53%; *p* = 0.042) (Figures 3F,G). Importantly, some MMR genes, such as *MLH3, MSH4, MSH3*, and *MLH1*, displayed high loss frequencies, which might diminish the expression of MMR genes and give rise to MSI in CRC.

### Methylation-Driven Genes

**Figure 4:**
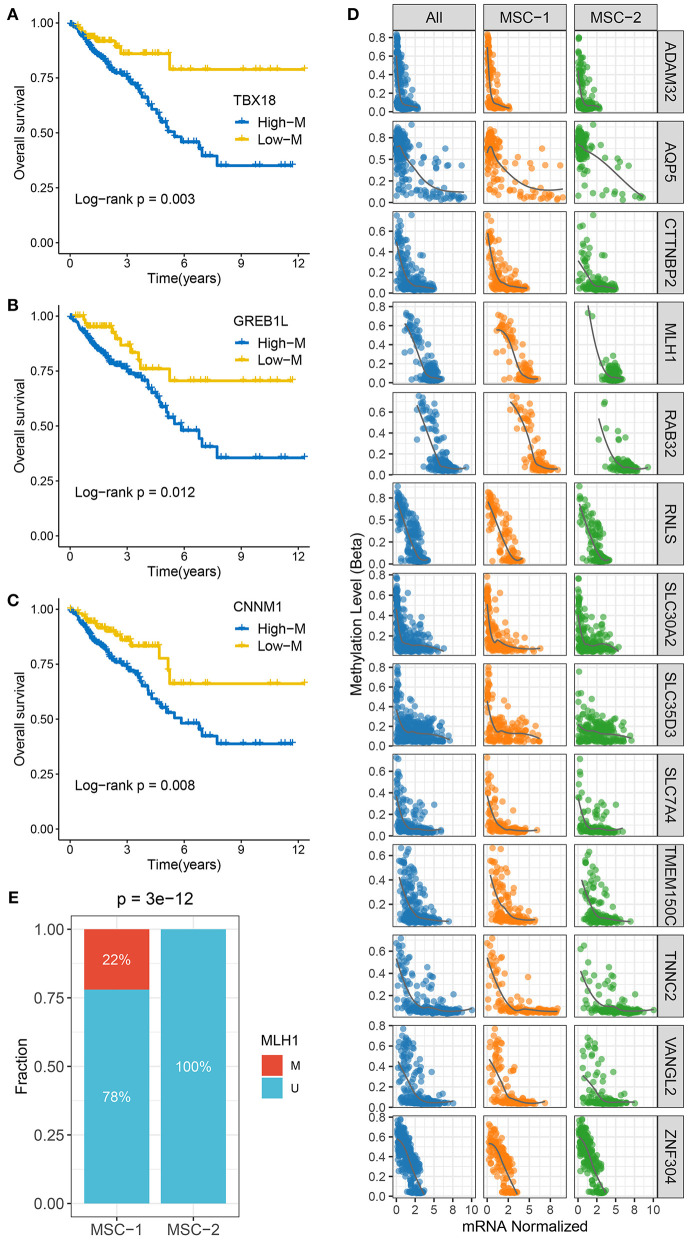
The methylation driven genes in CRC. **(A–C)** Kaplan–Meier survival analysis of *TBX18* **(A)**, *GREB1L* **(B)**, and *CNNM1* **(C)** methylation. **(D)** The correlation analysis between the methylation and mRNA expression levels of 13 ssMDGs. **(E)** The relative proportion of patients with the MMR genes methylation events between two subtypes.

To identify MDGs in CRC, the MethyMix package and the Wheeler criterion were employed. The MethyMix algorithm identified 608 genes with expression significantly related to methylation events, and the Wheeler criterion identified 147 epigenetically silenced genes (Supplementary Table 8). Ultimately, we identified a total of 69 MDGs by the intersection of the two methods. Further univariate Cox regression analysis uncovered the prognostic significance of these MSGs (Supplementary Table 9). High methylation of *TBX1, GREB1L*, and *CNNM1* was significantly associated with unfavorable OS (Figures 4A–C). Further investigation revealed that the high methylation of TBX18, GREB1L, and CNNM1 was still associated with adverse prognosis in MSC-1, while there was no significant correlation between the high methylation of CTNNB1 and OS in MSC-2 (Supplementary Figure 5A). In addition, we defined ssMDGs, and 13 ssMDGs had significantly different expression and methylation between the two MSC subtypes (Supplementary Figures 5B,C). For these ssMDGs, we observed a significant negative correlation between the expression and methylation levels (Figure 4D). MSC-2 featured more hypermethylation of *AQP5* and *ZNF304* than MSC-1. Interestingly, *AQP5* is a potential epigenetic driver of tumor development (42). The other 11 ssMDGs, such as *ADAM32, SLC35D3*, and *TMEM150C*, were specific for MSC-1. Of note, *MLH1* was also a specific ssMDG of MSC-1. As illustrated, the methylation level of *MLH-1* in MSC-1 was much higher than that in MSC-2, and the expression level was lower in MSC-1 than in MSC-2 (Supplementary Figures 5B,C). A previous report demonstrated that the hypermethylation of *MLH-1* was a potential mechanism contributing to MSI in CRC (43). We thus divided CRC patients into methylated cases and unmethylated cases based on a threshold of beta = 0.3 and found that all methylated cases were in MSC-1 (22 vs. 0%; *p* < 0.001) (Figure 4E), which explains the specificity of the MSI-associated mutational signature for MSC-1 to some extent.

### Functional Status, Immune Cell Infiltration, and Immunogenicity Assessment

**Figure 5:**
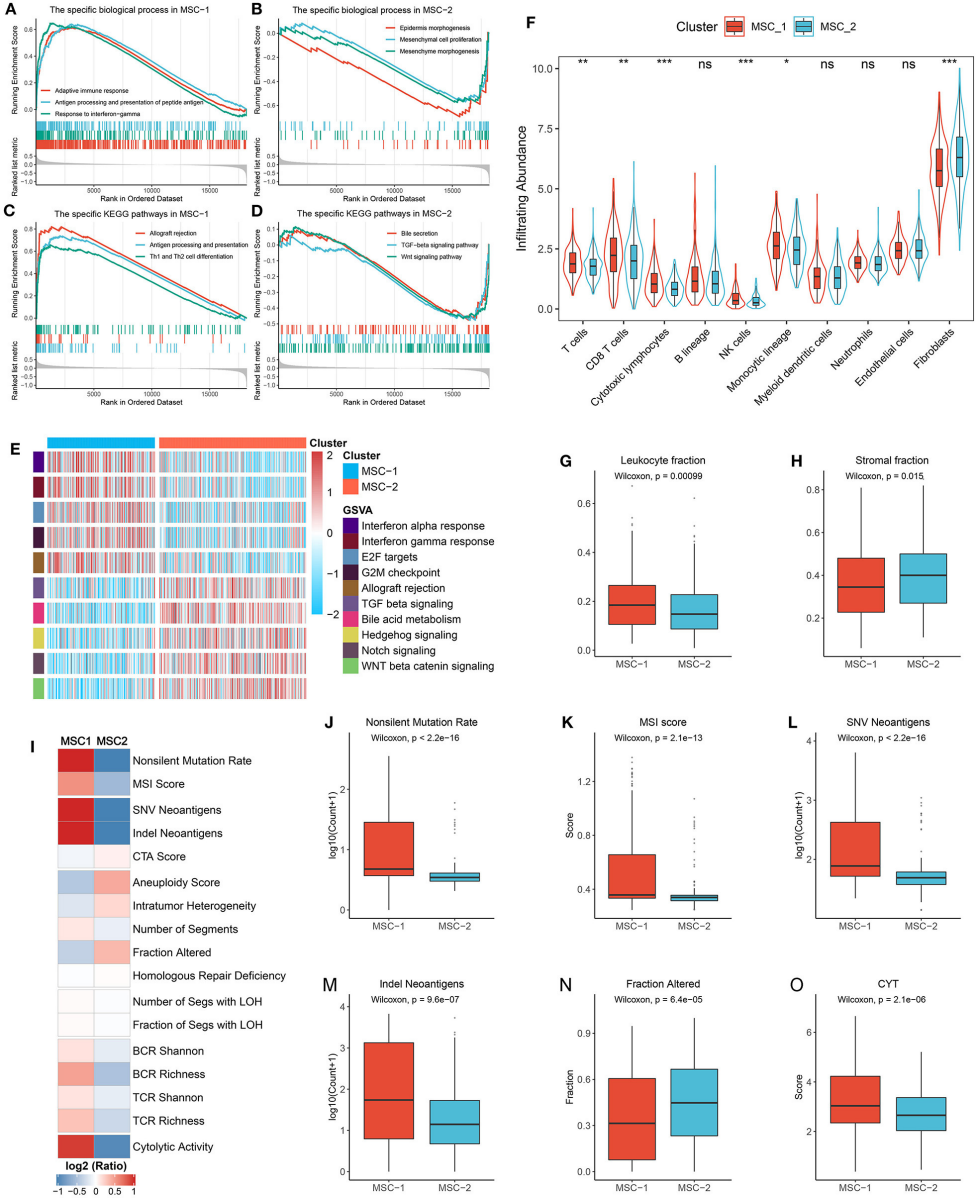
Functional status, immune cell infiltration, and immunogenicity assessment. **(A,B)** The biological process significantly enriched in MSC-1 **(A)** and MSC-2 **(B)**. **(C,D)** The KEGG pathways significantly enriched in MSC-1 **(C)** and MSC-2 **(D)**. **(E)** The specific Hallmark pathways in MSC-1 and MSC-2. **(F)** The infiltration abundance of eight immune cells and two non-immune cells populations in MSC-1 and MSC-2. ns, *P* > 0.05; *P < 0.05; **P < 0.01; ***P < 0.001. **(G,H)** The distribution of leukocyte **(G)** and stomal **(H)** fraction in MSC-1 and MSC-2. **(I)** The comparison of 17 immunogenicity associated indicators between two subtypes, the cell represented by the mean value of corresponding cluster divided by the overall mean value. **(J–O)** The distribution of non-silent mutation rate **(J)**, MSI score **(K)**, SNV neoantigens **(L)**, Indel neoantigens **(M)**, fraction of segments alteration **(N)**, and cytolytic activity (CYT) **(O)** in MSC-1 and MSC-2.

We performed biological process and KEGG pathway enrichment analyses through the GSEA approach. The MSC-1 subtype was tightly associated with immune-related pathways such as adaptive immune response, antigen processing and presentation, response to interferon-gamma, and Th1 and Th2 cell differentiation (Figures 5A,C). The MSC-2 subtype was significantly enriched in reactive stroma-related pathways such as epidermis or mesenchymal morphogenesis, mesenchymal cell proliferation, transforming growth factor beta (*TGF-*β) signaling, and Wnt signaling (Figures 5B,D). Further GSVA hallmark pathway assessment suggested a similar result to the above results and elucidated that MSC-1 showed predominant immune activation, such as activation of the canonical T-cell excitation molecule interferon-gamma, and MSC-2 showed obvious activation of stromal factors, such as *TGF-*β (Figure 5E). In addition, we also evaluated the difference in eight immune cell and two non-immune cell subpopulations between the two subtypes (Figure 5F). Consistently, cytotoxic immune cells, such as T-cells, CD8+ T-cells, cytotoxic lymphocytes, and natural killer cells, were found in higher proportions in MSC-1 than in MSC-2, while MSC-2 had higher proportions of fibroblasts. The leukocyte and stromal fraction data retrieved from Thorsson et al. (28) also demonstrated a dominant role of the MSC-1 subtype in immune activation and a dominant role of the MSC-2 subtype in stromal activation (Figures 5G,H).

Furthermore, 17 indicators were employed to deconvolute the immunogenicity features of the two subtypes (Supplementary Table 2, Figure 5I). In line with the specific mutational signatures in MSC-1 (that is, signatures 6, 10, and 15), the non-silent mutation rate and MSI score were higher in MSC-1 than in MSC-2 (Figures 5J,K). In addition, SNV and indels neoantigens were also more prone to occur in MSC-1 than in MSC-2 (Figures 5L,M), but there were no significant differences in terms of CTA score (Supplementary Figure 6A). Conversely, the CNV-relevant indicators, such as AS, ITH, number or fraction of altered segments, HRD, and LOH, were slightly higher in MSC-2 than in MSC-1, although most of the differences did not reach statistical significance (Figure 5N, Supplementary Figures 6B–G). These results imply that the immunogenicity of the two subtypes might be derived from their different genome alterations. In addition, the BCR/TCR diversity and CYT, which may reflect a robust antitumor response and cytolytic activity, were also higher in MSC-1 than in MSC-2 (Supplementary Figures 6H–K, Figure 5O). Overall, although there was heterogeneity between the two subtypes in different aspects of immunogenicity, MSC-1 still displayed stronger immunogenicity than MSC-2, and this increased immunogenicity might arise from the predominant mutation pattern. In addition, this stronger immunogenicity further conferred superior immune activation in MSC-1.

### The Expression and Regulation of Immune Checkpoint Molecules

**Figure 6:**
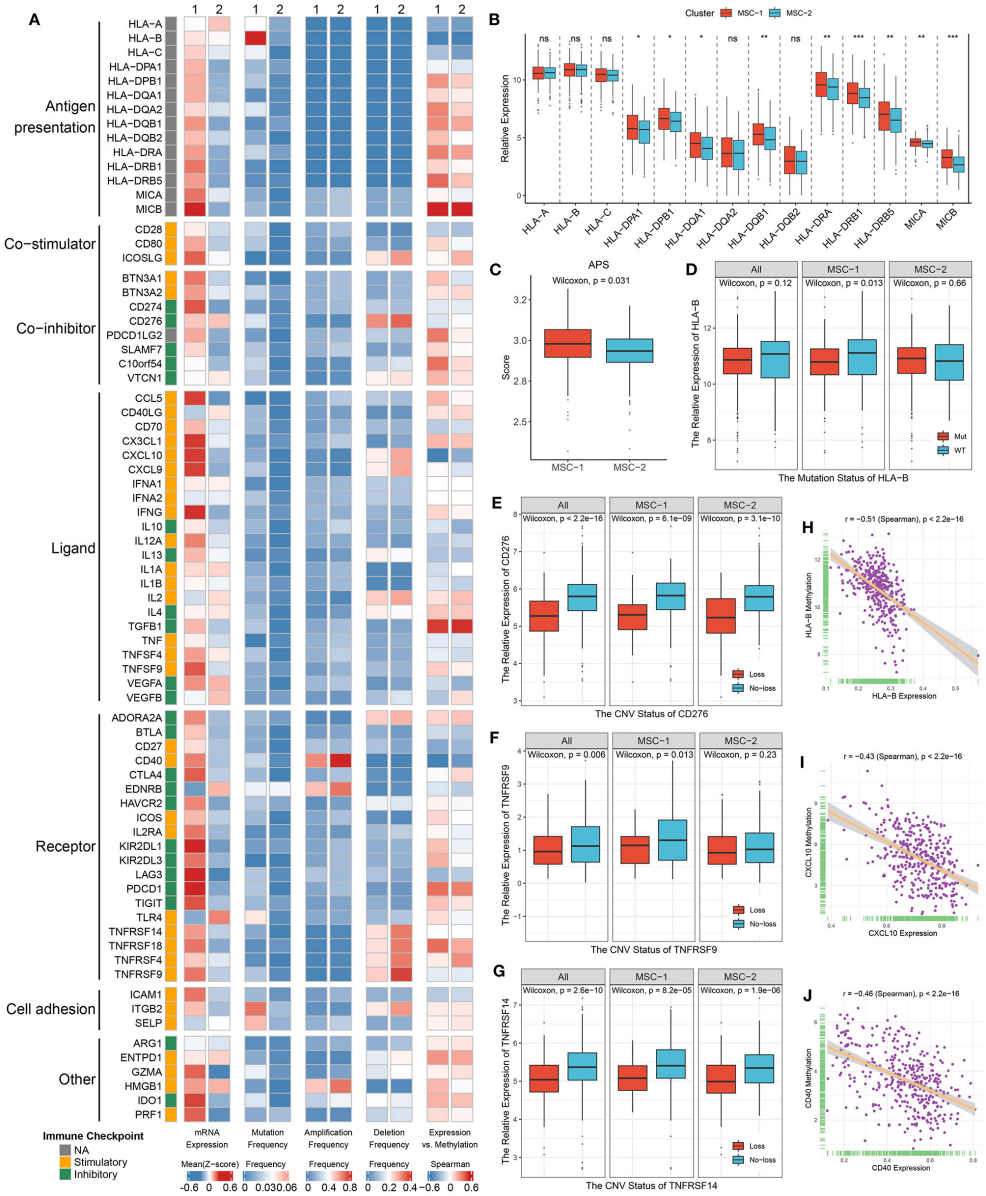
Multi-omics analysis of 75 immunomodulators in the TCGA-CRC cohort. **(A)** From left to right: mRNA expression (z-score), mutation frequency, amplification frequency, deletion frequency, and expression vs. methylation (gene expression correlation with DNA-methylation beta value) of 75 immunomodulators in MSC-1 and MSC-2. **(B)** The expression difference of MHC molecules between two subtypes. ns, *P* > 0.05; *P < 0.05; **P < 0.01; ***P < 0.001. **(C)** The distribution of APS score in MSC-1 and MSC-2. **(D–G)** The expression difference of HLA-B **(D)** between the mutant and wild groups, as well as *CD276* **(E)**, *TNFRSF9* **(F)**, and *TNFRSF14* **(G)** between loss and no-loss groups. **(H–J)** The correlation analysis between the methylation and mRNA expression levels of *HLA-B* **(H)**, *CXCL10* **(I)**, and *CD40* **(J)**.

We next explored the expression and regulation differences of 75 immune checkpoint molecules (ICMs) between the two MSC subtypes at the multiomics level (Supplementary Table 3). Obviously, the expression of ICMs was generally high in MSC-1 (Figure 6A, Supplementary Figures 7A,B). In addition, MHC molecules displayed relatively low expression in MHC-2 (Figure 6B). We further calculated the antigen processing and presenting machinery score (APS) via the ssGSEA algorithm and observed that MSC-2 also presented a lower APS than MSC-1 (Figure 6C). This suggested that antigen presentation capacity might be impaired. In line with the immune activation status, MSC-1 demonstrated higher expression of stimulatory ICMs such as *CCL5, CD40*, and *ITGB2* than MSC-2 (Supplementary Figure 7A). In addition, inhibitory ICMs such as *IDO1, PDCD1, CTLA4*, and *CD274* were also predominantly expressed in MSC-1, which implied that the overexpression of inhibitory ICMs might be a mechanism for immune escape in MSC-1 (Supplementary Figure 7B).

Furthermore, we integrated the mutation, SCNA, and methylation profiles to decipher the regulation of ICMs. Notably, although mutation of ICMs was generally rare (Figure 6A), it still induced some effects on the expression of ICMs; for example, mutation of *HLA-B* and *ITGB2* induced significantly lower expression in only MSC-1, but a slightly increased expression was observed in MSC-2 (Figure 6D, Supplementary Figure 7C). In contrast, SCNAs of ICMs were relatively prevalent (Figure 6A). *CD40* had the highest amplification frequency, but there was no significant expression difference between the gain and no-gain groups (Supplementary Figure 7D). Consistent with their deletion status, the expression of *CD276, ICOSLG, TNFRSF9, TNFRSF14*, and *TNRSF18* was relatively low in the loss group compared with the no-loss group (Figures 6E–G, Supplementary Figures 7E,F). In addition, hypermethylation also played a critical regulatory role for a number of ICMs, such as *HLA-B, CXCL10*, and *CD40*, and we observed that their expression was significantly negatively correlated with the methylation profile (*HLA-B*: r = −0.51; *CXCL10*: r = −0.43; *CD40*: r = −0.46; all *p* < 0.001) (Figures 6H–J).

### Identification of Reliable Gene Pair Markers for Predicting Prognosis and Distant Metastasis

**Figure 7:**
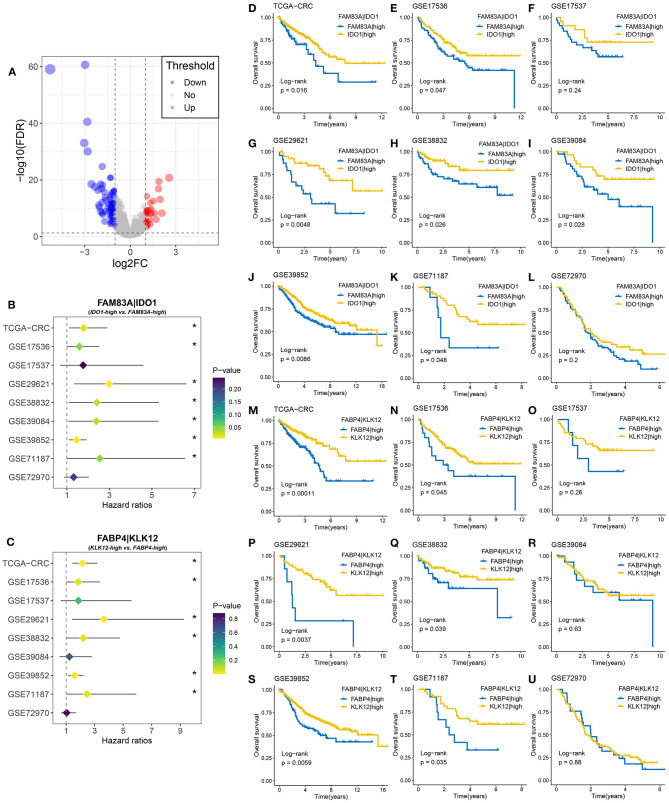
Identification of gene pairs with the ability to predict prognosis of CRC patients. **(A)** Volcano plot of differentially expressed genes (DEGs) between MSC-1 and MSC-2. The abscissa is log2FC, and the ordinate is –log10 (FDR). The red and blue points in the plot represent DEGs with statistical significance (FDR < 0.05 and |log2FC| > 1). **(B)** Forest plot of *IDO1*|high vs. *FAM83A*|high groups in nine cohorts. **(C)** Forest plot of *KLK12*|high vs. *FABP4*|high groups in nine cohorts. **(D–L)** Kaplan–Meier survival analysis for *FAM83A|IDO1* in the TCGA-CRC **(D)**, GSE17536 **(E)**, GSE17537 **(F)**, GSE29621 **(G)**, GSE38832 **(H)**, GSE39084 **(I)**, GSE39852 **(J)**, GSE71187 **(K)**, and GSE72970 **(L)** cohorts. **(M–U)** Kaplan–Meier survival analysis for *FABP4|KLK12* in the TCGA-CRC **(M)**, GSE17536 **(N)**, GSE17537 **(O)**, GSE29621 **(P)**, GSE38832 **(Q)**, GSE39084 **(R)**, GSE39852 **(S)**, GSE71187 **(T)**, and GSE72970 **(U)** cohorts. *P < 0.05.

A total of 108 DEGs were identified between the two MSC subtypes (Figure 7A, Supplementary Table 11). We further transformed the gene expression matrix into a two-gene expression relationship matrix. By using the pipeline to determine the consensus prognosis gene pair signature (CPGPS), we ultimately determined three gene pairs with dominant prognostic significance in at least five cohorts: *FAM83A|IDO1, FABP4|KLK12*, and *FABP4|GBP5* (Figures 7B,C, Supplementary Figure 7A). Of note, the gene pairs with a single relationship ratio in >90% of cases in the corresponding cohort were discarded. Ultimately, *FAM83A|IDO1* was removed based on the GSE103479 and GSE87211 cohorts, *FABP4|KLK12* was removed based on the GSE103479, GSE87211, GSE18105, GSE21510, GSE27854, and GSE71222 cohorts, and FABP4|GBP5 was removed based on the TCGA-CRC, GSE103479, GSE72970, GSE87211, GSE18105, GSE21510, GSE27854, and GSE71222 cohorts. The expression relationship of *FAM83A* and *IDO1* was significantly associated with prognosis in 7/9 cohorts (Figures 7B,D–L), and *FAM83A* > *IDO1* at the mRNA level was a poor prognostic factor. Although the *FAM83A*|high group had an adverse prognosis, there was no significance in the GSE17537 and GSE72970 cohorts, which might be due to their relatively small sample sizes (Figures 7F,L). The gene pair *FABP4|KLK12* was also a prognostic marker that exhibited significance in 6/9 cohorts. *FABP4* > *KLK12* was predominantly associated with unfavorable prognosis (Figures 7C,M–U). In addition, patients with *FABP4* > *GBP5* were more likely to have a poor prognosis than patients without this expression relationship in 5/7 cohorts (Supplementary Figures 8A–H). Further multivariate Cox analysis revealed that *FAM83A|IDO1* was an independent prognostic factor in most cohorts (7/9) (Supplementary Table 11). Conversely, the two gene pairs *FABP4|KLK12* and *FABP4|GBP5* did not perform well.

**Figure 8:**
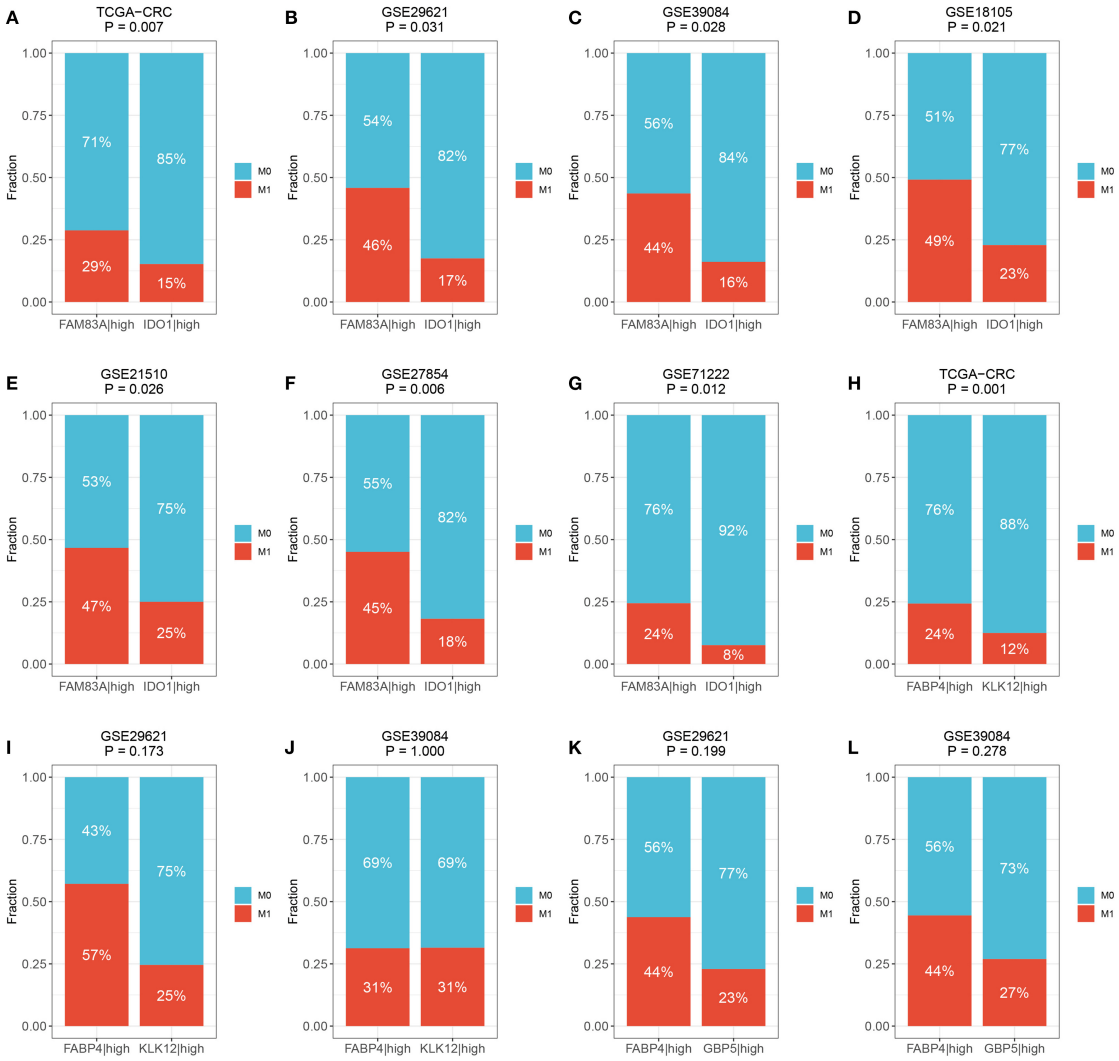
The predictive ability of three prognostic relevant gene pairs for distant metastasis. **(A–G)** The relative proportion of patients with distant metastasis between *FAM83A*|high and *IDO1*|high groups in the TCGA-CRC **(A)**, GSE29621 **(B)**, GSE39084 **(C)**, GSE18105 **(D)**, GSE27854 **(F)**, and GSE71222 **(G)** cohorts. **(H–J)** The relative proportion of patients with distant metastasis between *FABP4*|high and KLK12|high groups in the TCGA-CRC **(H)**, GSE29621 **(I)**, and GSE39084 **(J)** cohorts. **(K,L)** The relative proportion of patients with distant metastasis between FABP4|high and *GBP5*|high groups in GSE29621 **(K)** and GSE39084 **(L)** cohorts. M0, no metastasis; M1, metastasis.

We then determined the predictive role of the three gene pairs in CRC metastasis. Interestingly, the rate of distant metastasis was significantly different between the *FAM83A*|high and *IDO1*|high groups in all cohorts (TCGA-CRC: 29 vs. 15%, *p* = 0.007; GSE29621: 46 vs. 17%, *p* = 0.031; GSE39084: 44 vs. 16%, *p* = 0.028; GSE18105: 49 vs. 23%, *p* = 0.021; GSE21510: 47 vs. 25%, *p* = 0.026; GSE27854: 45 vs. 18%, *p* = 0.006; and GSE71222: 24 vs. 8%, *p* = 0.012) (Figures 8A–G). Due to the overrepresented single relationship of the gene pair, *FABP4|KLK12* and *FABP4|GBP5* were retained in three and two cohorts with metastasis information, respectively. Although statistical significance was not reached in most cohorts, the proportion of patients with metastasis varied between the *FABP4*|high and *KLK12|*high groups, and the *FABP4*|high and *GBP5*|high groups. For example, the *FABP4*|high group had a higher metastasis rate than the *KLK12*|high group (TCGA-CRC: 24 vs. 12%, *p* = 0.001; GSE29621: 57 vs. 25%, *p* = 0.173; and GSE39084: 31 vs. 31%, *p* = 1.000) (Figures 8H–J), and more metastatic cases were in the *FABP4*|high group than in the *GBP5*|high group (GSE29621: 44 vs. 23%, *p* = 0.199; and GSE39084: 44 vs. 27%, *p* = 0.278) (Figures 8K,L). Therefore, the predictive performance of *FABP4|KLK12* and *FABP4|GBP5* was much weaker than that of *FAM83A|IDO1*. Taken together, these data suggest that the expression relationships of *FAM83A* and *IDO1* are a very promising biomarker for predicting the prognosis and distant metastasis of CRC patients.

### Verification of the Role of FAM83A|IDO1 in Predicting Prognosis and Metastasis Using qRT-PCR

**Figure 9:**
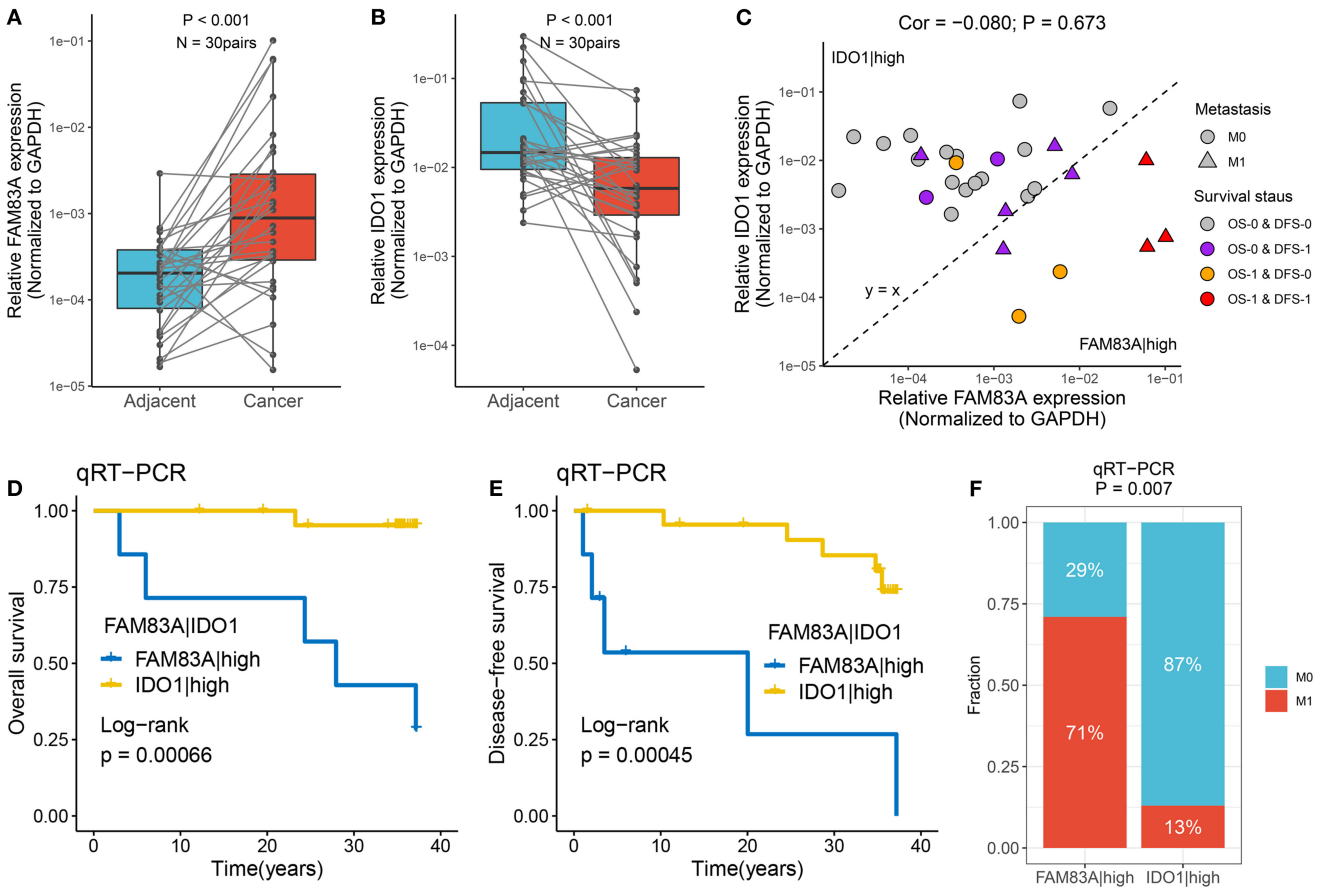
Verified the role of *FAM83A|IDO1* in prognosis and metastasis using qRT-PCR. **(A,B)** The expression difference of *FAM83A* **(A)** and *IDO1* **(B)** between two subtypes. **(C)** The mRNA expression of *FAM83A* and *IDO1* as well as the clinical outcomes in our cohort. The abscissa is the expression of *FAM83A*, and the ordinate is the expression of *IDO1*. Under the line y = x, *FAM83A* > *IDO1*, while above it, *FAM83A* < *IDO1*. M0, no metastasis; M1, metastasis. OS-0, alive; OS-1, death or censoring; DFS-0, disease free; DFS-1, disease or censoring. **(D,E)** Kaplan–Meier analysis of OS **(D)** and DFS **(E)** for *FAM83A|IDO1* in our cohort. **(F)** The relative proportion of patients with distant metastasis between *FAM83A*|high and *IDO1*|high groups in our cohort.

qRT-PCR assays were performed in 30 paired CRC tissues and matched adjacent non-tumor tissues (Supplementary Table 4). We observed that *FAM83A* was overexpressed in tumor tissues relative to adjacent non-tumor tissues, and the expression of *IDO1* showed the opposite relationship (*p* < 0.001) (Figures 9A,B). The role of *FAM83A|IDO1* in predicting prognosis and metastasis was further explored by qRT-PCR. The clinical outcome details (including survival status and metastasis status) of 30 CRC patients are shown in Figure 9C. There was no correlation between the expression of *FAM83A* and *IDO1*. In line with the previous results, when the expression of *FAM83A* was higher than that of *IDO1*, patients had worse OS and DFS (log-rank *p* < 0.001) (Figures 9D,E), as well as a stronger tendency for distant metastasis (71 vs. 13%, *p* = 0.007) (Figure 9F).

## Discussion

Elegant efforts have demonstrated that multifarious genomic alterations are critical to the prognosis and targeted therapy of CRC (44–48). We sought to better delineate the molecular diversity of CRC by determining mutational signatures that reflect different mutational processes, such as spontaneous deamination of 5-methylcytosine (signature 1), defective DNA MMR (signatures 6, 15, and 20), recurrent *POLE* somatic mutations (signature 10), and tobacco chewing habits (signature 29). Based on these signatures, we identified two heterogeneous subtypes, MSC-1 and MSC-2. The two subtypes exhibited tremendous differences in genomic alterations including mutation, SCNA, and DNA methylation. The distinct tumor microenvironment (TME) statuses and immune escape mechanisms of the two subtypes reinforced their molecular variability. We also observed significant clinical differences between the subtypes in terms of OS and DFS. In addition, to facilitate clinical application, we employed a gene pair pipeline to determine a prognostic and distant metastasis biomarker, *FAM83A|IDO1*, and further validated it in 15 independent datasets and qRT-PCR data from 30 samples.

MSC-1, a mutation-dominant subtype characterized by signatures 6, 15, and 20, was linked to defective DNA MMR. In line with this, MSC-1 harbored mutations in many drivers, such as *ATM, BRAF*, and *HMCN1*, which play vital roles in the tumorigenesis and development of many cancers (49). Previous studies have indicated that *ATM* is involved in cell cycle regulation and DNA damage recognition and repair and might increase cell resistance to cisplatin (50). Approximately 10% of patients with metastatic CRC possess the *BRAF* V600 mutation, which is related to poor prognosis (51). Interestingly, specific commutations, including *BRAF-HMCN* and *DNAH17-MDN1*, appeared only in MSC-1, which suggested that these specific commutations could be promisingly employed to distinguish different subtypes. In addition, a multitude of methylation drivers, such as *ADAM32, MLH-1*, and *CTTNBP2*, were significantly epigenetically silenced in MSC-1. Interestingly, methylation silencing of *MLH-1*, which has been reported to contribute to oncogenesis in CRC by activating the serrated neoplasia pathway, appeared in only MSC-1 (52). We speculate that activation of the serrated neoplasia pathway in combination with *BRAF* mutation might be important in MSC-1 tumorigenesis.

MSC-2, a CNA-dominant subtype characterized by signatures 1 and 29, was related to spontaneous deamination of 5-methylcytosine and tobacco chewing habits. MSC-2 displayed loss of *MYC* (8q24.21), *SMAD4* (18q21.2), and *PTEN* (10q23.31) as well as gain of *CCND3* (6p21.1) and *ERBB2* (17q12). The oncogene *ERBB2* has been shown to be amplified or overexpressed in multiple cancers, including colon cancers (53, 54). As reported, *ERBB2* amplification is an emerging therapeutic target and may also be a negative predictor of response to anti-EGFR therapy in CRC (55). Another promising candidate is *SMAD4*, a tumor suppressor that is the central node in *TGF-*β signaling (56). Studies have demonstrated that the loss of *SMAD4* is associated with poor prognosis and predisposition to chemoresistance, such as resistance to 5-fluorouracil, leucovorin, and irinotecan (57). Of note, although MSC-2 demonstrated lower TMB than MSC-1, the frequency of mutations in the drivers *APC* and *KRAS*, which occur early in the progression from colorectal adenoma to malignant carcinoma, were highest in MSC-2 (52). As reported, ~85% of CRC cases are thought to evolve from conventional adenomas with the acquisition of mutations in *APC, SMAD4, TP53, KRAS*, or *PI3KCA*, resulting in Wnt-β-catenin and *TGF-*β pathway activation; this process is referred to as the adenoma-to-carcinoma sequence. The above analysis suggests that the conventional adenoma-to-carcinoma sequence may be an important process of oncogenesis in MSC-2.

In the present study, we also assessed the differences in immune cell proportions, stromal cell infiltration, and immune escape mechanisms between the two subtypes. Consistent with the high mutation load in MSC-1, there was also infiltration of numerous innate and adaptive immune cells in the TME, which was linked to the immune inflammation status. The TME results were also supported by the finding of activation of immune-related pathways, including pathways related to the adaptive immune response, antigen processing and presentation of peptide antigens, and the response to interferon-gamma. In CRC, immune checkpoint inhibitors have been proven effective in heavily mutated tumors with MMR defects or high levels of MSI (3), which implies that patients in MSC-1 may benefit more from immunotherapy than patients in MSC-2. Although accompanied by both MSI and immune activation, MSC-1 exhibited unfavorable OS and DFS. High levels of immunosuppressive molecules in the TME may trigger immune resistance and escape mechanisms in MSC-1. Compared with MSC-1, MSC-2 was characterized by more fibroblasts and a lack of adaptive immune cells, accompanied by stromal-associated pathway activation, such as activation of pathway related to epidermis or mesenchymal morphogenesis, mesenchymal cell proliferation, *TGF-*β signaling, and Wnt signaling. Combined with this weaker immunogenicity, the insufficient immune cell infiltration in MSC-2 contributes to immune escape, suggesting that patients with the MSC-2 subtype might exhibit an unfavorable response to immunotherapy. Therefore, comprehensive analysis of molecular and immune microenvironment variability might contribute to optimizing the treatment and clinical management of CRC patients.

In addition, we comprehensively revealed many prognosis-relevant genomic events. In this study, we observed that mutation of *EYS*, as well as gain of *MLST8* and *MAP2K2*, could prolong OS, while mutation of *USH2*, loss of *DKK1, APC, MCC*, and *SMAD4*, and methylation of *TBX1* were linked to unfavorable prognosis. In addition, the prognostic value of some commutations was revealed for the first time. Commutation of *APC-TP53* demonstrated favorable DFS, and commutation of *APC-KRAS, KRAS-TP53*, and *KRAS-SYNE1* was significantly associated with poor DFS. Importantly, to facilitate clinical application, we identified three gene pairs with prognostic significance, *FAM83A|IDO1, FABP4|KLK12*, and *FABP4|GBP5*. *FAM83A|IDO1* was best at predicting prognosis in 11 public datasets and our own cohort, and it was an independent prognostic factor for CRC. *FAM83A|IDO1* also exhibited excellent performance in assessing the distant metastasis status in seven public datasets and our own cohort. Patients with *FAM83A*|high disease had a higher risk of metastasis than patients with *IDO1*|high disease. Traditionally, the batch effects of different platforms and the different cutoff values severely limit the clinical translation and application of biomarkers. In this study, we only focused on the mathematical relationship between the mRNA expression of two genes, which completely ignores the batch effects among different platforms and does not require definition of a cutoff value; it is just a binary relationship. Therefore, the relationship between *FAM83A* and *IDO1* mRNA expression is a promising biomarker for predicting prognosis and metastasis in clinical applications.

Our study also has a few limitations. First, it analyzed multidimensional data of genomic alterations in CRC but lacked microscopic experimental verification. Second, the identification of the relationship between *FAM83A* and *IDO1* mRNA expression as a biomarker focused on the mathematical relationship between two genes, but the biological relationship was not studied.

We described a novel molecular classification method for categorizing CRC into two clusters, suggesting intertumoral molecular variability. The two subtypes displayed distinct genomic drivers, prognoses, functional statuses, immune microenvironments, and MSI statuses, and targeting these differences might advance precise treatment and clinical management in CRC. Promisingly, we also identified and validated a robust and promising biomarker for predicting the prognosis and metastasis of CRC patients.

## Data Availability Statement

The raw data supporting the conclusions of this article will be made available by the authors, without undue reservation.

## Ethics Statement

The studies involving human participants were reviewed and approved by the human cancer tissues used in this study were approved by Ethnics Committee of The First Affiliated Hospital of Zhengzhou University in December 19, 2019, and the TRN is 2019-KW-423. The patients/participants provided their written informed consent to participate in this study.

## Author Contributions

ZLiu made the conceptualization. ZS, QD, and XH were involved in the methodology. ZLiu, DJ, ZLi, and XH provided the resources. ZLiu and QD analyzed the data. ZLiu, YZ, and KW prepared the original draft and reviewed and edited the manuscript. KW supervised the study. All authors contributed to the article and approved the submitted version.

## Conflict of Interest

The authors declare that the research was conducted in the absence of any commercial or financial relationships that could be construed as a potential conflict of interest.

## Supplementary Material

Supplementary Figure 1 Somatic mutation landscape in the TCGA-CRC cohort.

Supplementary Figure 2 The extraction of mutation signatures and generation of the mutation signature relevant subtypes in CRC. **(A)** Combining the cophenetic correlation coefficients and RSS curve, it was decided that rank = 8 was optimal in NMF clustering analysis. **(B)** The correlation analysis of *de novo* mutational signatures and curated signatures in COSMIC using cosine similarity. The rows are *de novo* mutational signatures and the columns are curated signatures in COSMIC. **(C)** The cumulative distribution functions (CDF) of consensus matrix for each k (k = 2~9, indicated by colors). **(D)** Proportion of ambiguous clustering (PAC) score, a low value of PAC implies a flat middle segment, allowing conjecture of the optimal k (k = 2) by the lowest PAC. **(E)** Recommended number of clusters using 26 criteria of Nbclust package. **(F)** Kaplan–Meier analysis for DFS between MSC-1 and MSC-2 within 5 years. **(G)** The relative proportion of eight mutation signatures between MSC-1 and MSC-2.

Supplementary Figure 3 The mutation drivers and MMR genes in CRC. **(A)** The distribution of tumor mutation burden (TMB) between two subtypes. **(B)** The mutation co-occurrence and exclusive relationships of 28 candidate driven genes. Co-occurrence, green; Exclusion, brown. **(C)** Kaplan-Meier survival analysis of APC-TP53 co-occurrence. **(D)** Mutational oncoplot of nine MMR genes between two subtypes.

Supplementary Figure 4 The driven segments identified from GISTIC algorithm in CRC. **(A)** The distribution of gain and loss load in arm-level and focal-level. **(B)** Oncoplot for the CNA of 39 driver segments in two subtypes, including 14 amplification segments (orange) and 25 deletion segments (purple). **(C)** The expression difference of CNA relevant oncogenes and tumor suppressive genes between gain (red) and no-gain (blue) groups or between loss (dark blue) and no-loss (orange) groups. ns, *P* > 0.05; ^*^*P* < 0.05; ^***^*P* < 0.001. **(D,E)** Univariate Cox regression analysis of 16 CNA relevant oncogenes and tumor suppressive genes for OS **(D)** and DFS **(E)**. **(F)** Kaplan-Meier survival analysis of MAP2K2 gain, as well as CNTN6, DKK1, APC, MCC, and SMAD4 loss.

Supplementary Figure 5 The methylation driven genes in CRC. **(A)** Kaplan–Meier survival analysis of TBX18, GREB1L, and CNNM1 methylation in MSC-1 and MSC-2, respectively. **(B)** The expression difference of 13 ssMDGs between two subtypes. **(C)** The methylation difference of 13 ssMDGs between two subtypes. ns, *P* > 0.05; ^*^*P* < 0.05; ^**^*P* < 0.01; ^***^*P* < 0.001.

Supplementary Figure 6 The difference of 10 immunogenicity relevant indicators between two subtypes. **(A–K)** The distribution of 10 immunogenicity relevant indicators in two subtypes, including CTA score **(A)**, aneuploidy score **(B)**, intratumor heterogeneity **(C)**, number of segments **(D)**, HRD **(E)**, number of segments with LOH **(F)**, fraction of segments with LOH **(G)**, BCR Shannon **(H)**, BCR richness **(I)**, TCR Shannon **(J)**, and TCR richness **(K)**.

Supplementary Figure 7 The expression and regulation of immune checkpoint molecules (ICMs) in MSC-1 and MSC-2. **(A)** The expression difference of 37 stimulatory ICMs in two subtypes. **(B)** The expression difference of 23 inhibitory ICMs in two subtypes. ns, *P* > 0.05; ^*^*P* < 0.05; ^**^*P* < 0.01; ^***^*P* < 0.001. **(C)** The expression difference of ITGB2 between the mutation and wild groups. **(D)** The expression difference of CD40 between the gain and no-gain groups. **(E,F)** The expression difference of ITGB2 **(E)** and TNFRSF18 **(F)** between the loss and no-loss groups.

Supplementary Figure 8 The prognostic value of FABP4|GBP5 in seven cohorts. **(A)** Forest plot of GBP5-high vs. FABP4-high groups in seven cohorts. **(B–H)** Kaplan-Meier survival analysis of FABP4-high and GBP5-high in the GSE17536 **(B)**, GSE17537 **(C)**, GSE29621 **(D)**, GSE38832 **(E)**, GSE39084 **(F)**, GSE39852 **(G)**, and GSE71187 cohorts **(H)**. ^*^*P* < 0.05.

Supplementary Table 1 Basic information of datasets included in this study.

Supplementary Table 2 The details of indicators for the assessment of immunogenicity and antigen presentation capacity.

Supplementary Table 3 The details of 75 immunomodulator molecules.

Supplementary Table 4 The qRT-PCR results and prognosis information of 30 CRC patients in our cohorts.

Supplementary Table 5 The details of 28 mutation drivers.

Supplementary Table 6 The significant focal copy number amplification of CRC in the TCGA cohort.

Supplementary Table 7 The significant focal copy number deletion of CRC in the TCGA cohort.

Supplementary Table 8 The details of methylation driven genes (MDGs).

Supplementary Table 9 The univariate Cox regression of 69 methylation driven genes.

Supplementary Table 10 The results of differential expression analysis. ^*^Differentially expressed genes (DEGs) are marked in red.

Supplementary Table 11 The multivariate Cox regression results of nine cohorts. ^*^The gene pairs with *P* < 0.05 are marked in red.
