# Supplementary figures and images for: Genomic Alteration Characterization in Colorectal Cancer Identifies a Prognostic and Metastasis Biomarker: FAM83A|IDO1
Source: Front Oncol. 2021 Apr 20;11:632430. doi: 10.3389/fonc.2021.632430 (PMC8093579; doi:10.3389/fonc.2021.632430)

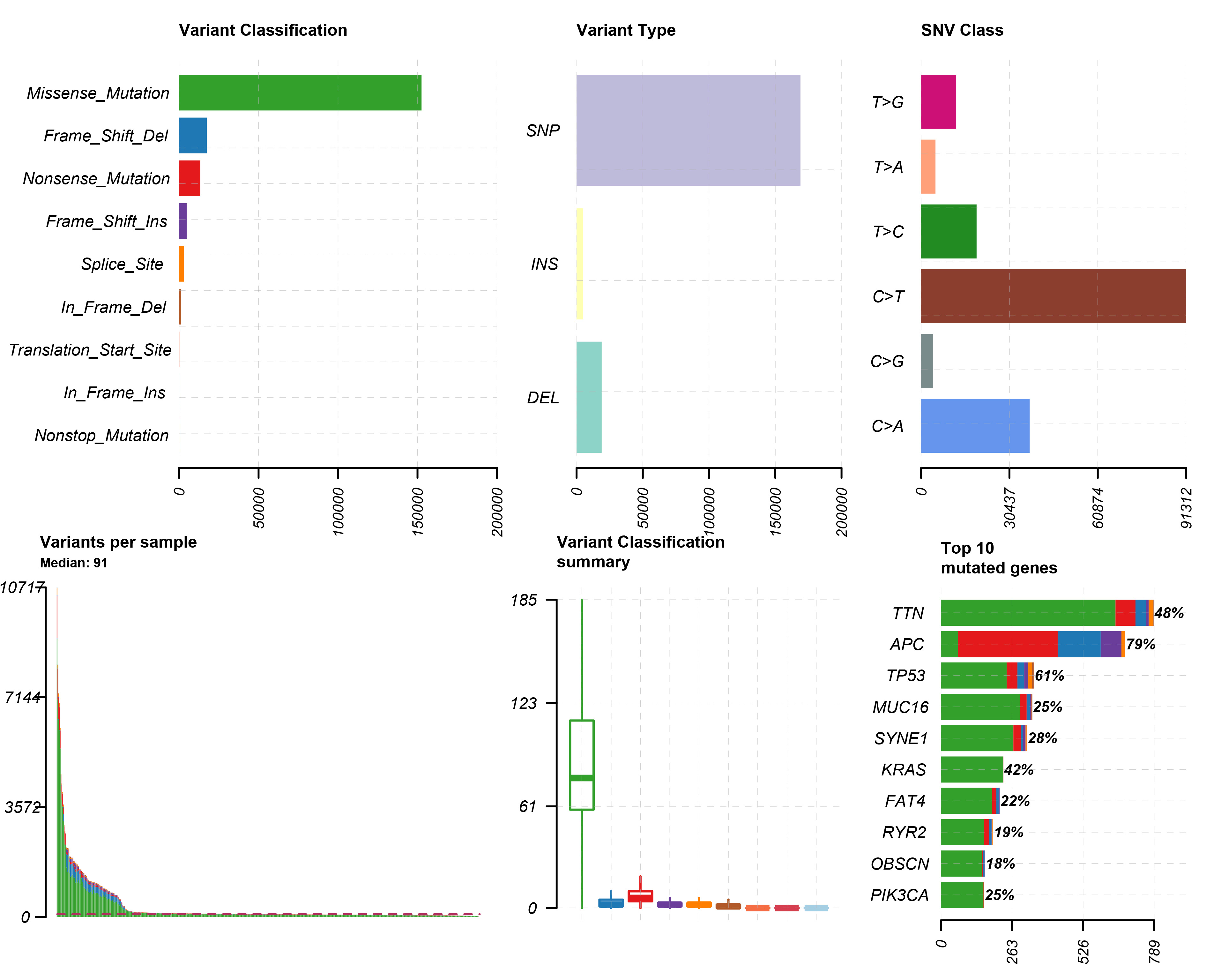

Supplement: Supplementary Figure 1 — Somatic mutation landscape in the TCGA-CRC cohort. [file Image_1.JPEG]

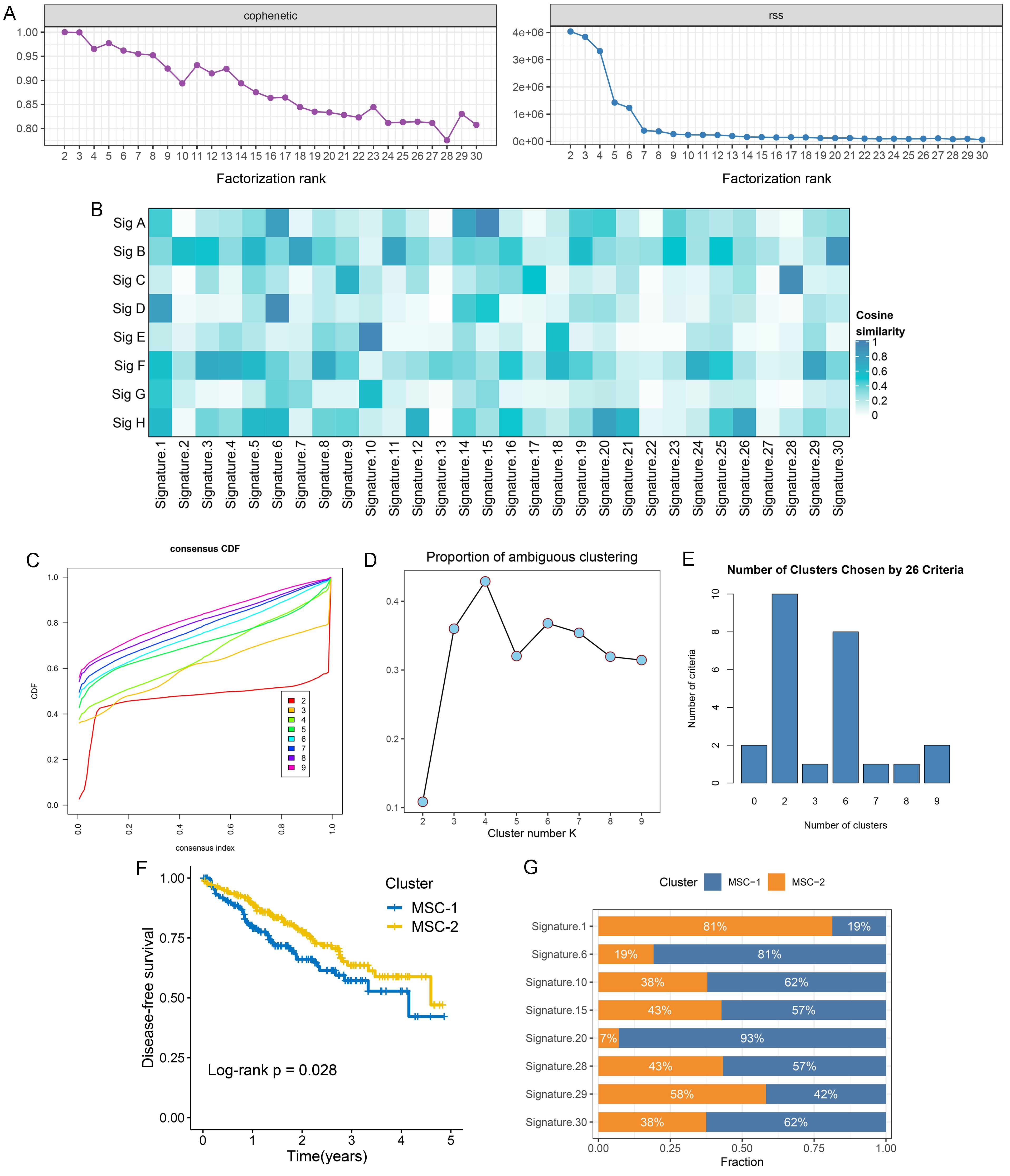

Supplement: Supplementary Figure 2 — The extraction of mutation signatures and generation of the mutation signature relevant subtypes in CRC. (A) Combining the cophenetic correlation coefficients and RSS curve, it was decided that rank = 8 was optimal in NMF clustering analysis. (B) The correlation analysis of de novo mutational signatures and curated signatures in COSMIC using cosine similarity. The rows are de novo mutational signatures and the columns are curated signatures in COSMIC. (C) The cumulative distribution functions (CDF) of consensus matrix for each k (k = 2~9, indicated by colors). (D) Proportion of ambiguous clustering (PAC) score, a low value of PAC implies a flat middle segment, allowing conjecture of the optimal k (k = 2) by the lowest PAC. (E) Recommended number of clusters using 26 criteria of Nbclust package. (F) Kaplan–Meier analysis for DFS between MSC-1 and MSC-2 within 5 years. (G) The relative proportion of eight mutation signatures between MSC-1 and MSC-2. [file Image_2.JPEG]

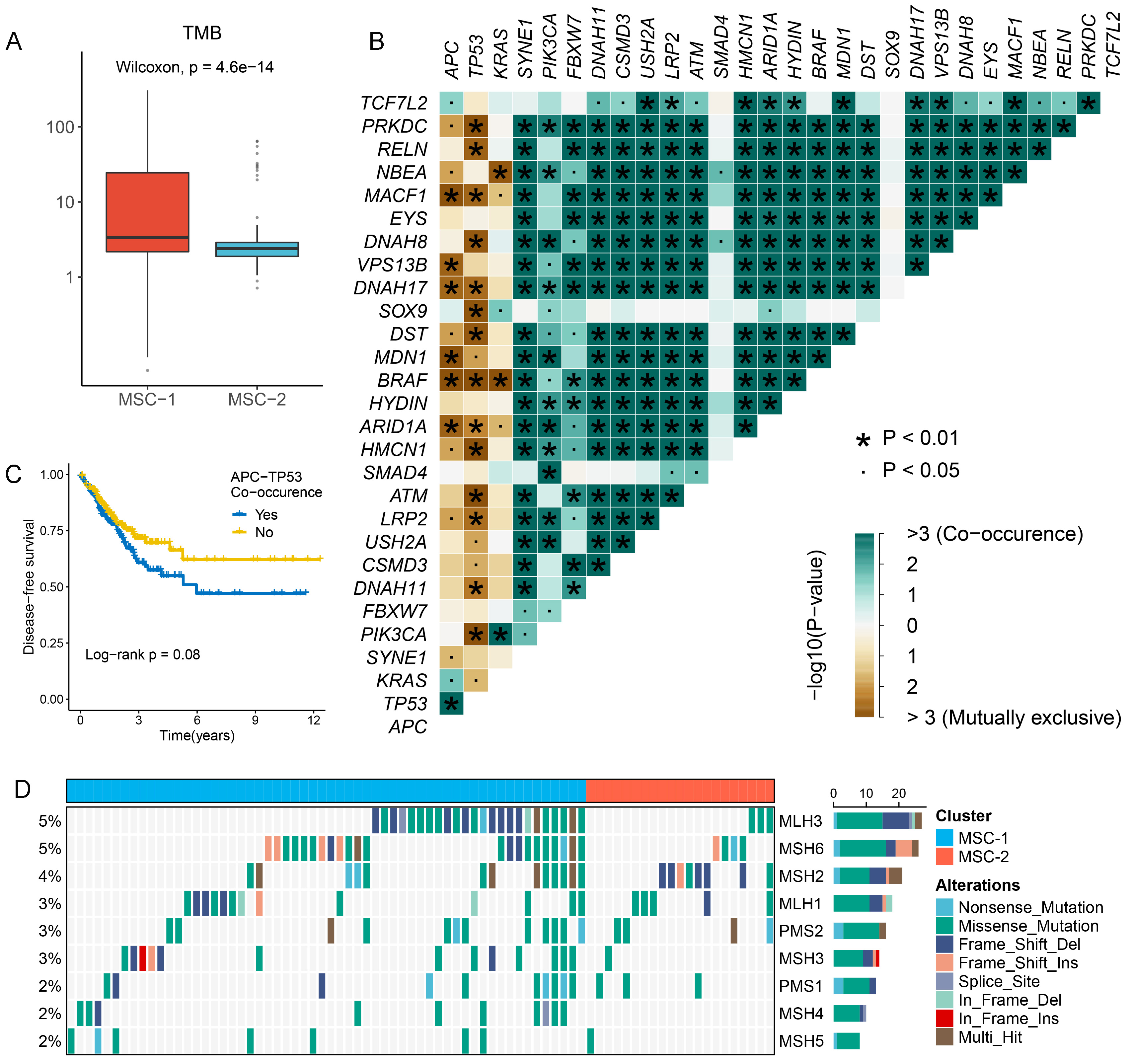

Supplement: Supplementary Figure 3 — The mutation drivers and MMR genes in CRC. (A) The distribution of tumor mutation burden (TMB) between two subtypes. (B) The mutation co-occurrence and exclusive relationships of 28 candidate driven genes. Co-occurrence, green; Exclusion, brown. (C) Kaplan-Meier survival analysis of APC-TP53 co-occurrence. (D) Mutational oncoplot of nine MMR genes between two subtypes. [file Image_3.JPEG]

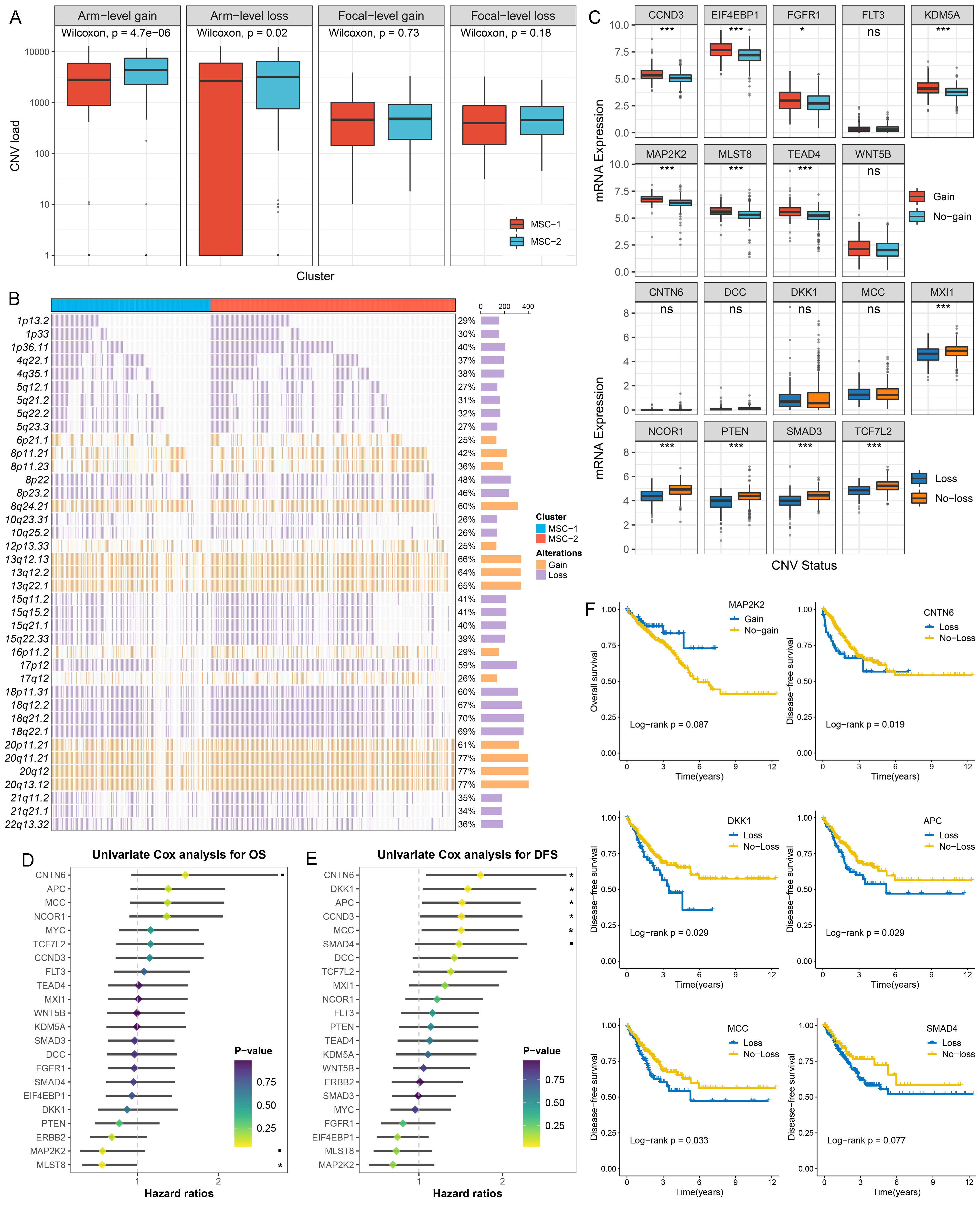

Supplement: Supplementary Figure 4 — The driven segments identified from GISTIC algorithm in CRC. (A) The distribution of gain and loss load in arm-level and focal-level. (B) Oncoplot for the CNA of 39 driver segments in two subtypes, including 14 amplification segments (orange) and 25 deletion segments (purple). (C) The expression difference of CNA relevant oncogenes and tumor suppressive genes between gain (red) and no-gain (blue) groups or between loss (dark blue) and no-loss (orange) groups. ns, P > 0.05; *P < 0.05; ***P < 0.001. (D,E) Univariate Cox regression analysis of 16 CNA relevant oncogenes and tumor suppressive genes for OS (D) and DFS (E). (F) Kaplan-Meier survival analysis of MAP2K2 gain, as well as CNTN6, DKK1, APC, MCC, and SMAD4 loss. [file Image_4.JPEG]

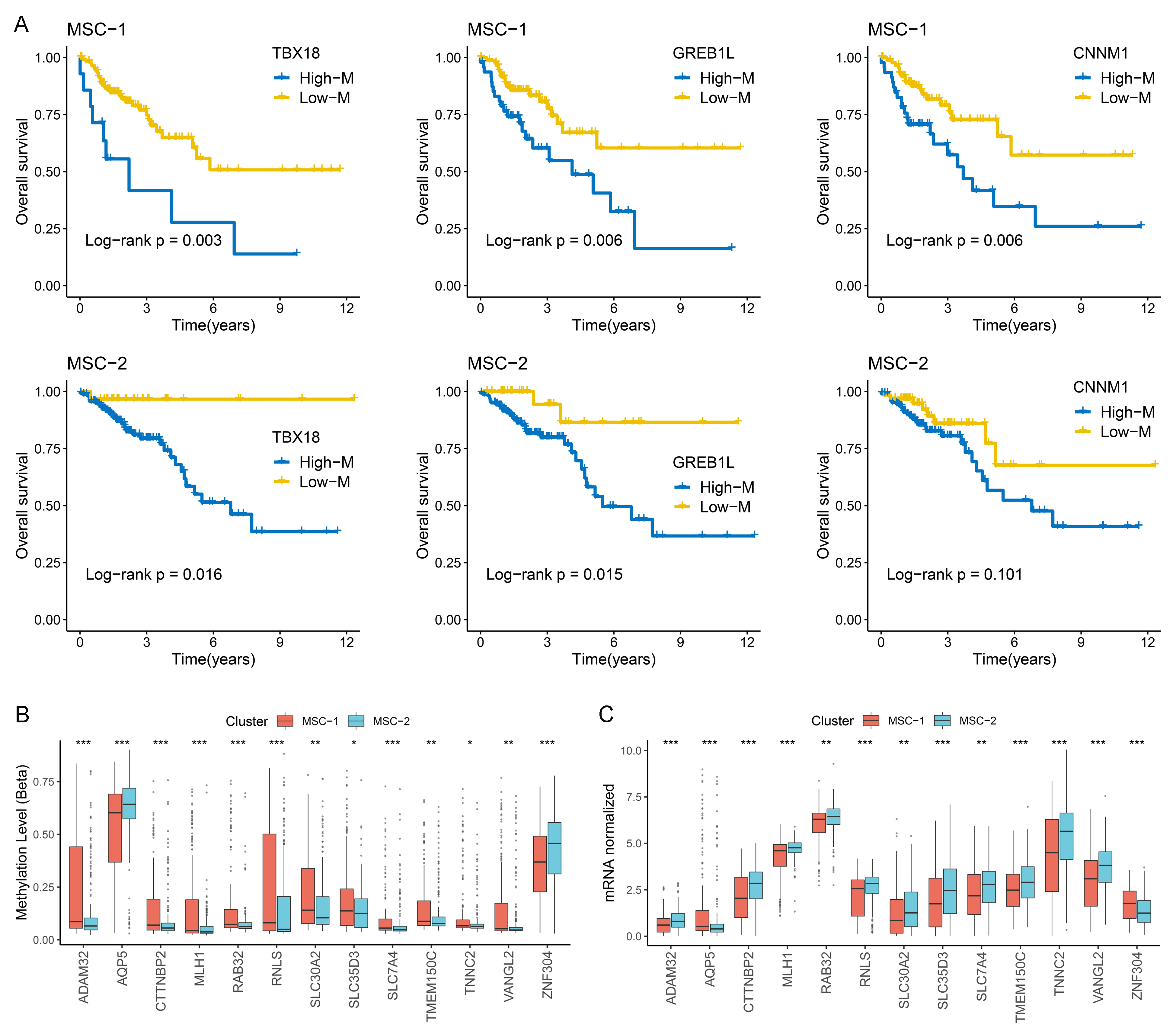

Supplement: Supplementary Figure 5 — The methylation driven genes in CRC. (A) Kaplan–Meier survival analysis of TBX18, GREB1L, and CNNM1 methylation in MSC-1 and MSC-2, respectively. (B) The expression difference of 13 ssMDGs between two subtypes. (C) The methylation difference of 13 ssMDGs between two subtypes. ns, P > 0.05; *P < 0.05; **P < 0.01; ***P < 0.001. [file Image_5.JPEG]

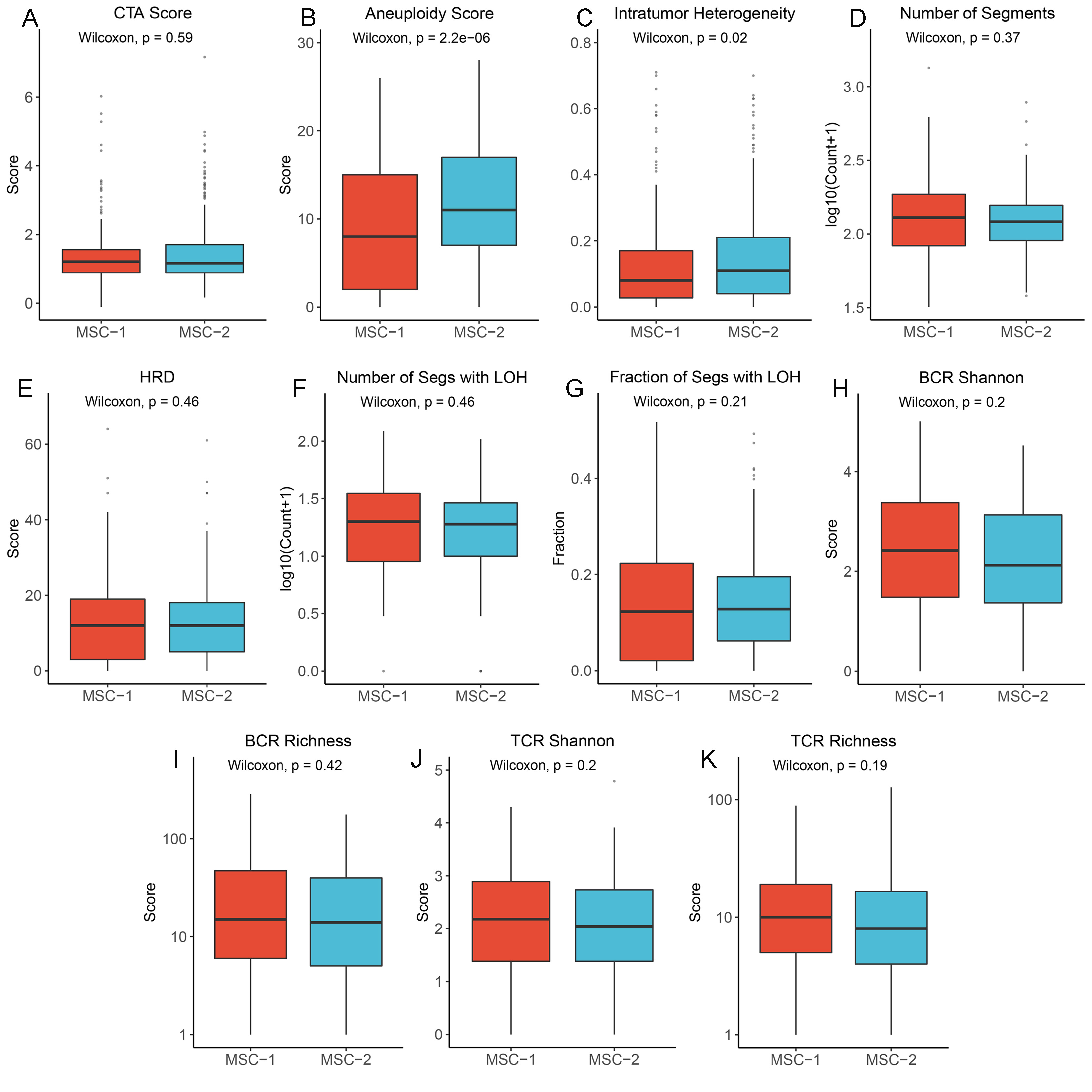

Supplement: Supplementary Figure 6 — The difference of 10 immunogenicity relevant indicators between two subtypes. (A–K) The distribution of 10 immunogenicity relevant indicators in two subtypes, including CTA score (A), aneuploidy score (B), intratumor heterogeneity (C), number of segments (D), HRD (E), number of segments with LOH (F), fraction of segments with LOH (G), BCR Shannon (H), BCR richness (I), TCR Shannon (J), and TCR richness (K). [file Image_6.JPEG]

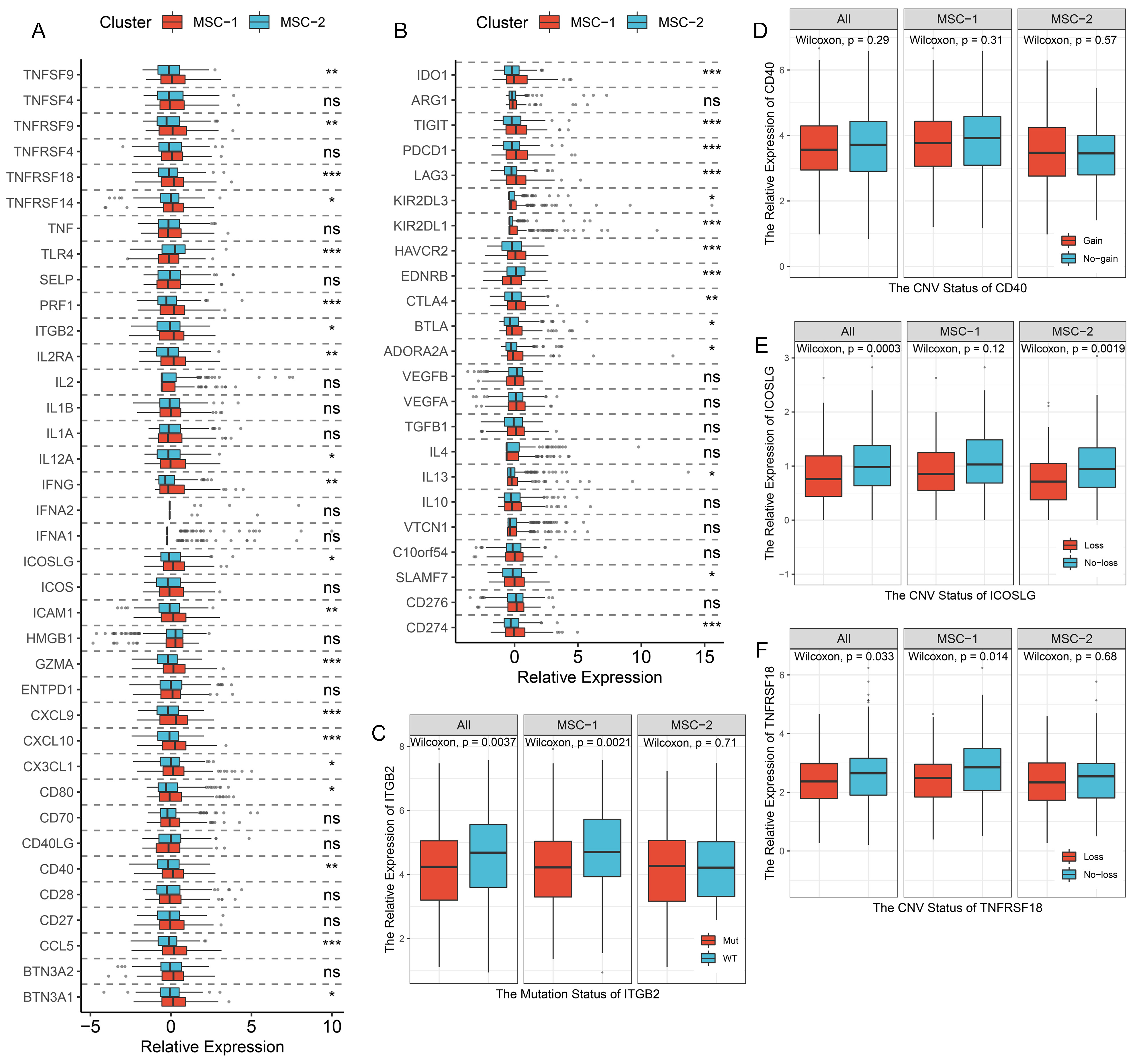

Supplement: Supplementary Figure 7 — The expression and regulation of immune checkpoint molecules (ICMs) in MSC-1 and MSC-2. (A) The expression difference of 37 stimulatory ICMs in two subtypes. (B) The expression difference of 23 inhibitory ICMs in two subtypes. ns, P > 0.05; *P < 0.05; **P < 0.01; ***P < 0.001. (C) The expression difference of ITGB2 between the mutation and wild groups. (D) The expression difference of CD40 between the gain and no-gain groups. (E,F) The expression difference of ITGB2 (E) and TNFRSF18 (F) between the loss and no-loss groups. [file Image_7.JPEG]

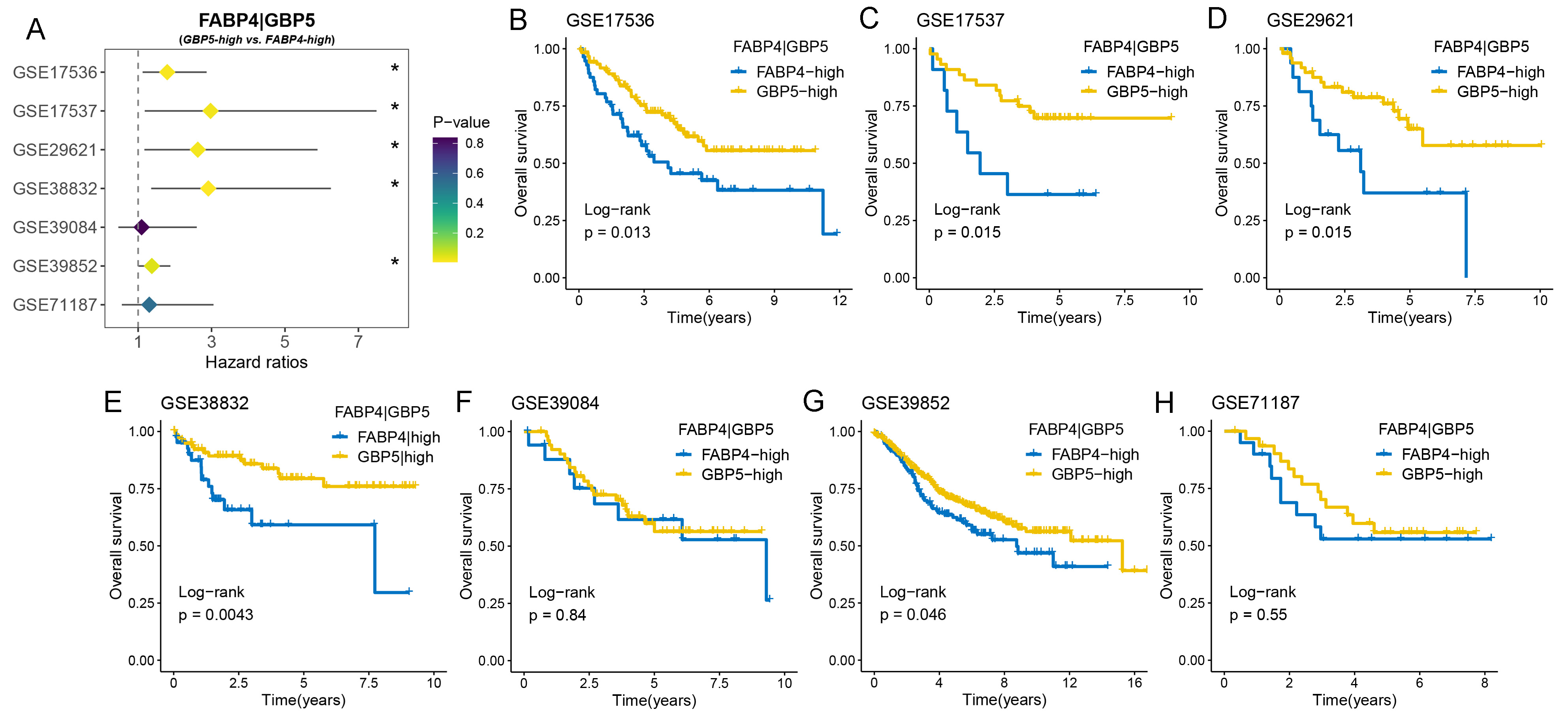

Supplement: Supplementary Figure 8 — The prognostic value of FABP4|GBP5 in seven cohorts. (A) Forest plot of GBP5-high vs. FABP4-high groups in seven cohorts. (B–H) Kaplan-Meier survival analysis of FABP4-high and GBP5-high in the GSE17536 (B), GSE17537 (C), GSE29621 (D), GSE38832 (E), GSE39084 (F), GSE39852 (G), and GSE71187 cohorts (H). *P < 0.05. [file Image_8.JPEG]
